# Differential roles of eNOS in late effects of VEGF-A on hyperpermeability in different types of endothelial cells

**DOI:** 10.1038/s41598-023-46893-4

**Published:** 2023-12-05

**Authors:** Esmeralda K. Bosma, Shahan Darwesh, Yasmin I. Habani, Maxime Cammeraat, Paola Serrano Martinez, Mathilda E. van Breest Smallenburg, Jia Y. Zheng, Ilse M. C. Vogels, Cornelis J. F. van Noorden, Reinier O. Schlingemann, Ingeborg Klaassen

**Affiliations:** 1 Ocular Angiogenesis Group, Department of Ophthalmology, Amsterdam UMC Location University of Amsterdam, Meibergdreef 9, Amsterdam, The Netherlands; 2 Amsterdam Cardiovascular Sciences, Microcirculation, Amsterdam, The Netherlands; 3 Amsterdam Neuroscience, Cellular & Molecular Mechanisms, Amsterdam, The Netherlands; 4 Department of Genetic Toxicology and Cancer Biology, National Institute of Biology, Ljubljana, Slovenia; 5 Department of Ophthalmology, University of Lausanne, Jules Gonin Eye Hospital, Fondation Asile Des Aveugles, Lausanne, Switzerland

**Keywords:** Endocytosis, RNAi

## Abstract

Vascular endothelial growth factor (VEGF)-A induces endothelial hyperpermeability, but the molecular pathways remain incompletely understood. Endothelial nitric oxide synthase (eNOS) regulates acute effects of VEGF-A on permeability of endothelial cells (ECs), but it remains unknown whether and how eNOS regulates late effects of VEGF-A-induced hyperpermeability. Here we show that VEGF-A induces hyperpermeability via eNOS-dependent and eNOS-independent mechanisms at 2 days after VEGF-A stimulation. Silencing of expression of the eNOS gene (*NOS3*) reduced VEGF-A-induced permeability for dextran (70 kDa) and 766 Da-tracer in human dermal microvascular ECs (HDMVECs), but not in human retinal microvascular ECs (HRECs) and human umbilical vein ECs (HUVECs). However, silencing of *NOS3* expression in HRECs increased permeability to dextran, BSA and 766 Da-tracer in the absence of VEGF-A stimulation, suggesting a barrier-protective function of eNOS. We also investigated how silencing of *NOS3* expression regulates the expression of permeability-related transcripts, and found that *NOS3* silencing downregulates the expression of *PLVAP*, a molecule associated with trans-endothelial transport via caveolae, in HDMVECs and HUVECs, but not in HRECs. Our findings underscore the complexity of VEGF-A-induced permeability pathways in ECs and the role of eNOS therein, and demonstrate that different pathways are activated depending on the EC phenotype.

## Introduction

Endothelial transcytosis is an important transport pathway over the vascular wall, which together with paracellular passage contributes to vascular permeability. Vascular endothelial growth factor-A (VEGF-A) induces vascular hyperpermeability in specific tissues and in various pathological conditions, including diabetic retinopathy and ischemic disorders^1–6^. Endothelial nitric oxide synthase [eNOS; also known as nitric oxide synthase 3 (NOS3)] is known to regulate the acute effects of VEGF-A on vascular hyperpermeability, as inhibition of eNOS expression or activity reduces VEGF-A-induced leakage^7–10^.

eNOS has a reductase domain and an oxygenase domain, and functions as dimer or monomer^11^. Dimeric eNOS synthesizes nitric oxide (NO) and l-citrulline from l-arginine and O_2_. Monomeric eNOS is uncoupled from NO production and produces the toxic metabolite superoxide (O_2_^−·^), which may react with NO to form peroxynitrite (ONOO^−·^). NO is a short-lived signaling molecule, and acts via its main receptor soluble guanylate cyclase (sGC) to produce the second messenger cyclic guanosine monophosphate (cGMP). Its long-term effects are mediated via the production of redox metabolites or modifications of protein thiols (S-nitrosylation) and tyrosines (tyrosine nitration)^12^. eNOS activity is tightly controlled by post-translational modifications and protein–protein interactions^13^.

eNOS-derived NO regulates acute effects of VEGF-A on paracellular transport by disassembly of adherens junction complexes in human and rodent endothelial cells (ECs)^7, 10, 14–16^. In addition, eNOS regulates early effects of VEGF on transcellular caveolar transport in cultured bovine retinal ECs (BRECs)^17^. However, the effects of VEGF-A on permeability are biphasic, with rapid transient effects, followed by sustained delayed effects^18^. In fact, effects of VEGF-A on endothelial permeability can be detected days after treatment in both in vivo and in vitro models^19–23^. For instance, repeated VEGF injections in monkey eyes promote leakage of retinal blood vessels at day 8 and 12 as determined with fluorescein angiography, increase the number of pinocytotic caveolar vesicles and alter the distribution of these vesicles in retinal ECs^19^. Moreover, VEGF-A increases the expression of the caveolae-associated protein plasmalemma vesicle-associated protein (PLVAP) up to 6 days after VEGF-A stimulation in cultured immortalized BRECs^22^ and in retinal blood vessels of monkeys at day 9 after multiple VEGF injections^24^. PLVAP has been shown to regulate VEGF-A-induced hyperpermeability at 3 days after VEGF-A stimulation, probably by regulating caveolar vesicle numbers^21^. Presently, the function of eNOS in the regulation of late effects of VEGF-A on endothelial hyperpermeability remains to be elucidated.

The aim of the present study was to investigate the role of eNOS in endothelial hyperpermeability at 2 days after VEGF-A stimulation, focusing on transcellular permeability. Experiments were performed in four different EC phenotypes, which are characterized by differences in species and tissue of origin, vascular caliber, physiological characteristics and intrinsic barrier properties. We included two types of human microvascular ECs derived from skin and retina, of which the latter are characterized by specialized blood-retinal barrier properties, and the commonly used macrovascular human umbilical vein ECs (HUVECs). In order to broaden our understanding of the function of eNOS in endothelial hyperpermeability, some experiments were repeated in BRECs.

## Results

### eNOS regulates late effects of VEGF-A on endothelial hyperpermeability in HDMVECs, but not in HRECs or HUVECs

**Figure 1:**
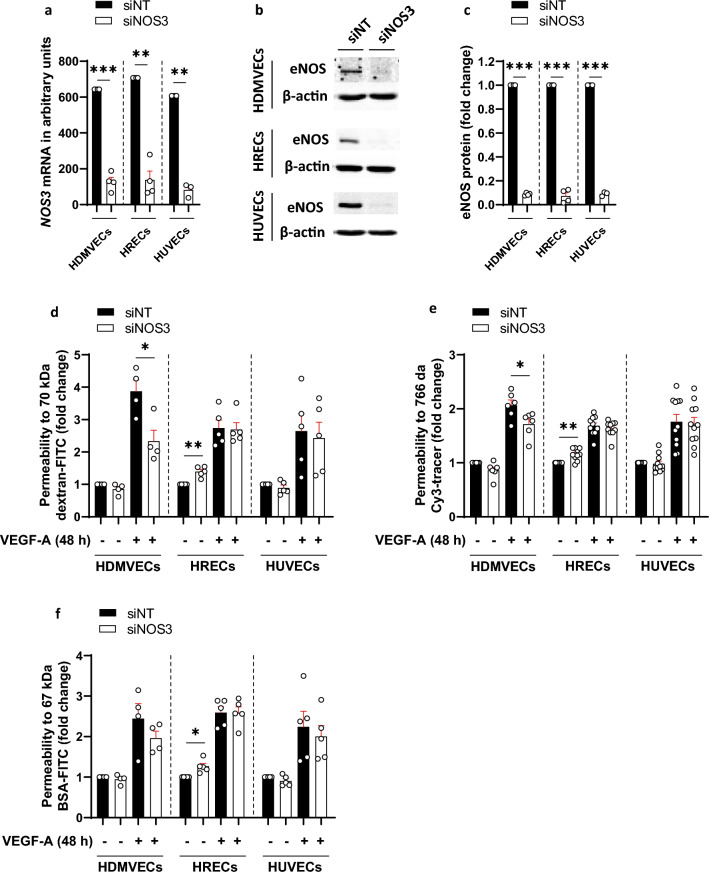
VEGF-A-induced hyperpermeability is partially facilitated by eNOS in HDMVECs, but not in HRECs and HUVECs. (**a**) Relative *NOS3* mRNA levels in control and si*NOS3*-treated HDMVECs, HRECs and HUVECs at 72 h after transfection, in *n* = 3–4 independent experiments. (**b**) Representative cropped images of western blots of eNOS and β-actin (loading control) expression in HDMVECs, HRECs and HUVECs transfected with siNT or si*NOS3*. Original blots are presented in Supplementary Figs. S1–S3. (**c**) eNOS protein levels were significantly reduced in si*NOS3*-treated HDMVECs, HRECs and HUVECs at 72 h after siRNA transfection, in *n* = 3–4 independent experiments. Permeability for 70 kDa dextran-FITC (**d**), 766 Da Cy3 tracer (**e**) and 67 kDa BSA-FITC (**f**) in siNT- and si*NOS3*-treated HDMVECs, HRECs and HUVECs in the presence and absence of exogenous basolateral VEGF-A (25 ng/mL) stimulation. *n* ≥ 4 independent experiments (each dot represents 1 experiment). Results are normalized to the unstimulated siNT control. Data are represented as mean ± SEM. *p < 0.05; **p < 0.01; ***p < 0.001 (one-sample t-test or Student’s t-test).

First, we determined whether eNOS is involved in the regulation of late effects of VEGF-A on endothelial hyperpermeability. Therefore, we silenced eNOS mRNA (*NOS3*) expression in human dermal microvascular ECs (HDMVECs), human retinal microvascular ECs (HRECs) and HUVECs with small interfering RNA (siRNA). Silencing of *NOS3* expression resulted in significantly decreased *NOS3* mRNA levels and eNOS protein levels at 72h after transfection as compared to non-targeting siRNA (siNT)-treated cells (Fig. 1a–c). Reduced *NOS3* expression levels were maintained up to 96 h post-transfection (Supplementary Fig. S4a). The expression of *NOS1* and *NOS2* was negligible or absent in our ECs. Silencing of *NOS3* expression did not alter the expression of *NOS1* and *NOS2* in most human ECs, except for HUVECs, where it resulted in upregulation of *NOS2* expression (Supplementary Fig. S4b,c).

VEGF-A induced permeability of tracers in all three human EC types (Fig. 1d–f). However, silencing of *NOS3* expression significantly attenuated VEGF-A-induced permeability of 70 kDa dextran-FITC and 766 Da Cy3-tracer in HDMVECs (by 1.7-fold and 1.2-fold, respectively), whereas this effect was not observed in HRECs and HUVECs (Fig. 1d,e). In contrast, silencing of *NOS3* expression did not alter VEGF-A-induced permeability of 67 kDa BSA-FITC in HDMVECs, HRECs and HUVECs (Fig. 1f). A similar trend on VEGF-A-induced tracer permeability was detected when *NOS3* expression was silenced in HDMVECs cultured in different medium and with different coatings, suggesting that the responses were specific for HDMVECs and not dependent on culture conditions (Supplementary Fig. S5). Silencing of *NOS3* expression in HRECs in the absence of VEGF-A stimulation resulted in increased permeability for dextran-FITC, BSA-FITC and Cy3-tracer (by 1.4-fold, 1.3-fold and 1.2-fold, respectively), whereas these responses were not detected in HDMVECs and HUVECs (Fig. 1d–f).

In conclusion, these findings indicate that eNOS partially increases the late effects of VEGF-A on endothelial hyperpermeability in HDMVECs, but not in HRECs and HUVECs. On the other hand, eNOS protects the endothelial barrier function in unstimulated HRECs, but not in unstimulated HDMVECs and HUVECs.

**Figure 2:**
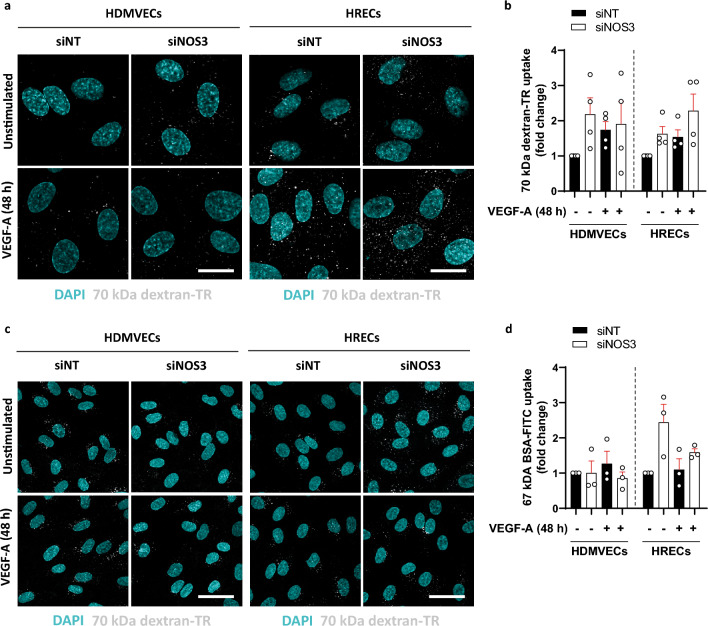
Effect of *NOS3* silencing on tracer uptake in HDMVECs and HRECs. (**a**) Representative images of dextran-Texas Red uptake in HDMVECs and HRECs treated with siNT and si*NOS3*. Cells were left unstimulated or treated with VEGF-A (25 ng/mL) for 48 h prior to the addition of the tracer. Quantification of dextran uptake is shown in (**b**), in *n* = 4 independent experiments. (**c**) Representative images of BSA-FITC uptake in HDMVECs and HRECs treated with siNT and si*NOS3*. (**d**) Quantification of BSA-FITC uptake in images shown in (**d**), *n* = 3 independent experiments. All results are normalized to the unstimulated siNT control. Scale bars: 20 µm. Data are represented as mean ± SEM.

Increased tracer leakage across a monolayer of cells in Transwell permeability experiments can be caused by either increased endothelial transcytosis or paracellular leakage, or both. To study whether eNOS alters VEGF-A-induced transcellular transport, we investigated whether eNOS regulates tracer uptake, which is the first critical step in transcytosis. Silencing of *NOS3* expression did not alter the uptake of 70 kDa dextran-Texas Red and 67 kDa BSA-FITC in the absence or presence of exogenous VEGF-A in HDMVECs and HRECs (Fig. 2). This suggests that the effects of silencing of *NOS3* expression on endothelial permeability are regulated during later steps of transcytosis or via paracellular permeability. However, it should be noted that there was a trend of increased tracer uptake in si*NOS3*-treated ECs.

### eNOS regulates the expression of PLVAP in HDMVECs and HUVECs

**Figure 3:**
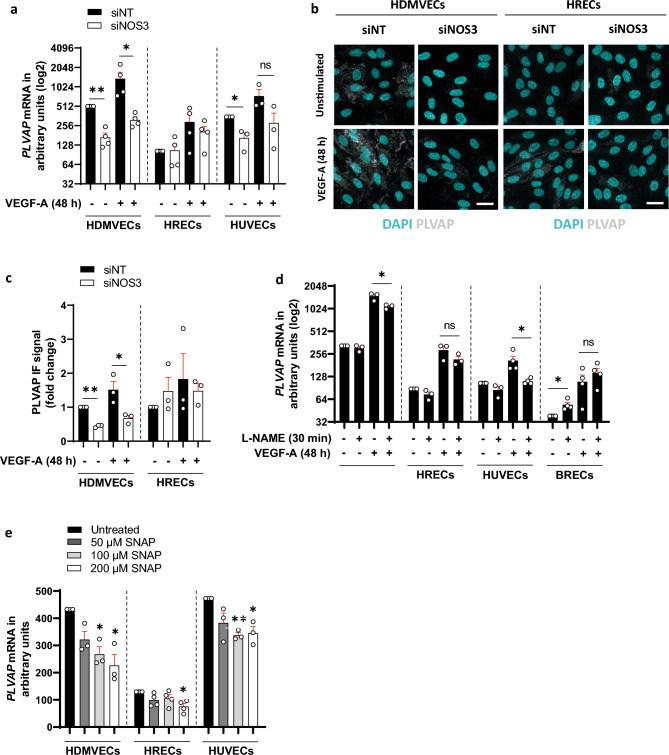
PLVAP expression is regulated by eNOS in HDMVECs and HUVECs, but not in HRECs. (**a**) *PLVAP* mRNA levels in VEGF-A-stimulated (25 ng/mL for 48 h) siNT- and si*NOS3*-treated HDMVECs, HRECs and HUVECs in *n* = 3–4 independent experiments. (**b**) Representative confocal images of PLVAP immunofluorescence staining in siNT- and si*NOS3*-treated HDMVECs and HRECs. Cells were cultured in the presence or absence of VEGF-A (25 ng/mL for 48 h). Scale bars: 20 µm. (**c**) Quantification of PLVAP immunofluorescence staining data shown in (**b**), *n* = 3 independent experiments. (**d**) *PLVAP* mRNA levels in control, L-NAME pretreated (100 µM, 30 min), VEGF-A-stimulated (25 ng/mL for 48 h) or a combination of L-NAME pretreated and VEGF-A-stimulated HDMVECs, HRECs, HUVECs and BRECs, in *n* = 3–4 independent experiments. (**e**) *PLVAP* mRNA levels in HDMVECs, HRECs and HUVECs after repeated treatment with NO donor SNAP (50 µM, 100 µM or 200 µM) over a 48 h incubation period (cells were stimulated 3 times for 4 h per day with freshly prepared SNAP), in *n* = 3–4 independent experiments. Data are represented as mean ± SEM. *p < 0.05; **p < 0.01; ***p < 0.001; ns, not significant (one-sample t-test or Student’s t-test).

It has been demonstrated that PLVAP plays an essential role in VEGF-A-induced hyperpermeability of large tracers^21^. Therefore, we analyzed how silencing of *NOS3* expression affects PLVAP expression. PLVAP is normally absent in blood-retinal barrier ECs in vivo^25–27^. However, in our cultured HRECs low levels of PLVAP on the mRNA and protein levels were found (Fig. 3a–c). In the absence of exogenous VEGF-A, silencing of *NOS3* expression downregulated mRNA expression of *PLVAP* in HDMVECs and HUVECs (by 3.0-fold and 2.0-fold, respectively), but not in HRECs (Fig. 3a). In the presence of exogenous VEGF-A, silencing of *NOS3* expression significantly downregulated mRNA expression of *PLVAP* in HDMVECs (by 4.3-fold). In HRECs, significant differences were not found, whereas there was only a non-significant trend towards lower *PLVAP* levels in HUVECs (Fig. 3a).

Next, we determined whether these transcriptional effects on PLVAP expression in HDMVECs and HRECs were reflected at the protein level. In line with the findings at the mRNA level, we found that silencing of *NOS3* expression reduced PLVAP protein expression in HDMVECs by 2.2-fold both in the presence and absence of exogenous VEGF-A, whereas this effect was not observed in HRECs (Fig. 3b,c).

To further investigate how eNOS regulates VEGF-A-induced PLVAP expression, we analyzed how the NOS inhibitor L-NAME affects VEGF-A-induced upregulation of *PLVAP* mRNA expression in ECs. For this purpose, cells were treated for 30 min with 100 µM L-NAME prior to VEGF-A stimulation according to Feng et al.^17^. In addition to the experiments with HDMVECs, HRECs and HUVECs, we also included BRECs of early passage in this analysis. Cultured BRECs express lower basal levels of *PLVAP* mRNA, and therefore better represent blood-retinal barrier ECs. Inhibition of eNOS activity with L-NAME dampened VEGF-A-induced upregulation of *PLVAP* mRNA expression in HDMVECs and in HUVECs (by 1.3-fold and 1.6-fold, respectively), but not in HRECs or BRECs (Fig. 3d). L-NAME pretreatment in the absence of VEGF-A stimulation increased mRNA expression of *PLVAP* in BRECs (by 1.4-fold), whereas this response was not detected in HDMVECs, HRECs and HUVECs (Fig. 3d). Taken together, these findings indicate that eNOS has an effect on VEGF-A-induced expression of *PLVAP* in HDMVECs and HUVECs, but not in HRECs and BRECs.

Dimeric eNOS produces NO, but uncoupled eNOS produces the toxic product O2^−·^, which may react with NO to generate ONOO^−·^^12^. Uncoupling occurs when NO levels increase and NO outcompetes superoxide dismutase, which detoxifies O2^−·^ radicals. During aging and in pathological conditions such as diabetes, ischemic disease and atherosclerosis, endothelial production of ONOO^−·^ is increased^28^. To investigate whether the effects of *NOS3* silencing on *PLVAP* expression are mediated by decreased NO levels or rather by a reduction of the levels of toxic products, we treated ECs with the NO donor SNAP for 48 h, and subsequently evaluated *PLVAP* mRNA expression. As the NO donor SNAP has a short half-life, we stimulated the cells 3 times for 4 h per day with freshly prepared SNAP. SNAP treatment downregulated *PLVAP* mRNA expression in HDMVECs (100 µM SNAP: 1.6-fold; 200 µM SNAP: 1.9-fold), HRECs (200 µM SNAP: 1.7-fold) and HUVECs (100 µM SNAP: 1.4-fold; 200 µM SNAP: 1.4-fold) (Fig. 3e). This shows that the NO donor SNAP lowers *PLVAP* expression in all three human EC types, suggesting that the observed responses in our si*NOS3*-treated cells may be caused by other reaction products of eNOS.

We investigated the functional role of PLVAP in HDMVECs by rescuing PLVAP expression in si*NOS3*-treated HDMVECs with lentiviral transduction, and performed permeability experiments with 70 kDa dextran-FITC and 766 Da Cy3-tracer. Overexpression of *PLVAP* did not change permeability of dextran-FITC and 766 Da Cy3-tracer in si*NOS3*-treated HDMVECs compared with siNT-treated HDMVECs, both in the presence and absence of exogenous VEGF-A (Supplementary Fig. S6).

### Silencing of *NOS3* expression alters VEGFR2 expression in HUVECs

**Figure 4:**
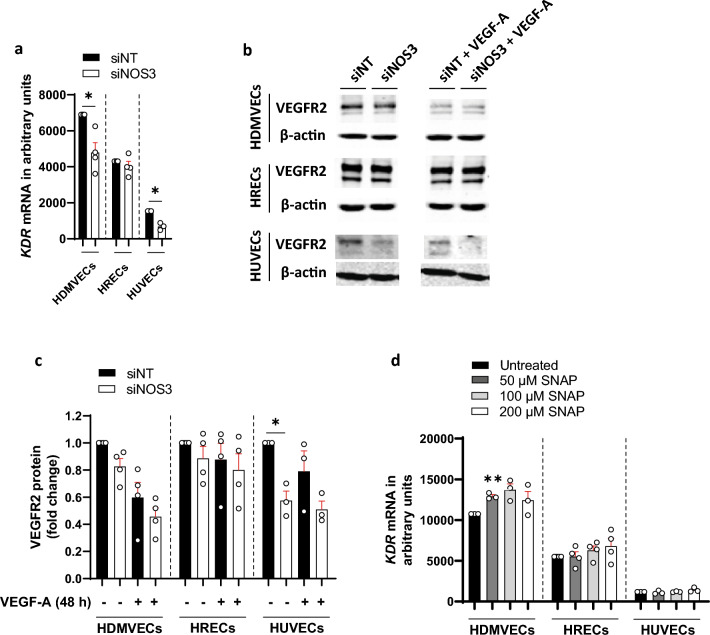
VEGFR2 expression is regulated by eNOS in HUVECs, but not in HDMVECs and HUVECs. (**a**) Relative *KDR* mRNA levels in control and si*NOS3*-treated HDMVECs, HRECs and HUVECs at 72 h after transfection, in *n* = 3–4 independent experiments. (**b**) Representative cropped images of western blots of VEGFR2 and β-actin (loading control) expression in HDMVECs, HRECs and HUVECs transfected with siNT or si*NOS3*. Cells were cultured in the presence or absence of VEGF-A (25 ng/mL for 48 h). Original blots are presented in Supplementary Figs. S2, S7 and S8. (**c**) VEGFR2 protein levels in control and in si*NOS3*-treated HDMVECs, HRECs and HUVECs at 72 h after siRNA transfection, in *n* = 3–4 independent experiments. (**d**) *KDR* mRNA levels in HDMVECs, HRECs and HUVECs after repeated treatment with NO donor SNAP (50 µM, 100 µM or 200 µM) over a 48 h incubation period (cells were stimulated 3 times for 4 h per day with freshly prepared SNAP), in *n* = 3–4 independent experiments. Data are represented as mean ± SEM. *p < 0.05; **p < 0.01 (one-sample t-test or Student’s t-test).

VEGF-A acts through VEGF receptor 2 (VEGFR2/*KDR*) to induce permeability^29^. Moreover, PLVAP mRNA and protein expression is regulated in a VEGF-A/VEGFR2-dependent manner^30^. To investigate how eNOS regulates VEGF-A-induced permeability, we determined how loss of eNOS expression affects the mRNA and protein expression of VEGFR2 in the three human EC subtypes. Silencing of *NOS3* expression lowered the expression of *KDR* in HDMVECs and in HUVECs (by 1.9-fold and 2.2-fold, respectively), but not in HRECs (Fig. 4a).

We subsequently analyzed how silencing of *NOS3* expression regulates VEGFR2 protein levels in all three human cell types. We found that silencing of *NOS3* expression did not significantly alter VEGFR2 protein levels in HDMVECs and HRECs both in the presence and absence of exogenous VEGF-A, whereas silencing of *NOS3* expression in HUVECs reduced VEGFR2 protein levels in the absence of exogenous VEGF-A (by 1.7-fold; Fig. 4b,c). Next, we analyzed whether treatment with the NO donor SNAP affects *KDR* expression in HDMVECs, HRECs and HUVECs. SNAP increased *KDR* expression by 1.2-fold when used at a concentration of 50 µM in HDMVECs, whereas no effect of SNAP treatment was detected when used at 100 µM and 200 µM concentration (Fig. 4d). In addition, SNAP treatment did not alter *KDR* expression in HRECs and HUVECs (Fig. 4d).

In conclusion, eNOS regulates VEGFR2 protein expression in HUVECs, but not in HDMVECs and HRECs. This suggests that the observed responses of silencing of *NOS3* expression on VEGF-A-induced endothelial hyperpermeability in HDMVECs are not regulated at the receptor level, but rather regulated via downstream effectors.

### Silencing of *NOS3* expression has limited or no effects on the expression of various transcellular transport-related, paracellular transport-related and VEGF family-related transcripts in HDMVECs, HRECs and HUVECs

Next, we analyzed whether silencing of *NOS3* expression regulates the expression of other permeability-related transcripts besides *PLVAP* and *KDR*. We analyzed how silencing of *NOS3* expression alters the expression of transcripts related to various aspects of transcytosis, including the formation and stability of various vesicle subtypes (*CAV1*, *CLTC*, *FLOT1*, *FLOT2*, *PACSIN2*, *MFSD2A*), endocytosis of vesicles (*DNM1*,* DNM2*), and docking and fusion of exocytotic vesicles (*NSF*, *SNAP23*, *VAMP2*, *VAMP3*, *VAMP8*). The function of these targets in transcellular transport are discussed in Table 1. In addition, we analyzed how silencing of *NOS3* expression affects the expression of adherens junctions (*CDH5*, *CTNNB1)*, tight junctions (*CLDN5*, *OCLN*, *TJP1)*, and VEGF family-related transcripts (*FLT1*, *FLT4*, *NRP1*, *NRP2*, *VEGFA*).

**Table 1:** Proteins and protein complexes involved in the regulation of transcellular transport in ECs.

Gene	Full description	Function	References
PLVAP	Plasmalemma vesicle-associated protein	Forms a molecular-sieve structure, also known as stomatal and fenestral diaphragms, on top of caveolae, fenestrae and transendothelial channels Regulates caveolar shape	^25, 26, 31, 32^
CAV1	Caveolin-1	Structural component of caveolae Regulates caveolae biogenesis	^33^
CLTC	Clathrin	Clathrin heavy chain and light chain assemble in a trimeric structure called triskelion, that constitutes the clathrin coat Clathrin-coated vesicles play a role in receptor-mediated uptake of molecules	^34, 35^
DNM1 or DNM2	Dynamin-1 or dynamin-2	Regulates membrane fission, necessary for endocytosis	^36^
FLOT1 or FLOT2	Flotillin-1 or flotillin-2	Are enriched in lipid rafts May induce membrane invaginations, reminiscent of caveolae May stabilize caveolin-1 levels by preventing lysosomal degradation	^37, 38^
MFSD2A	Major facilitator superfamily domain containing 2a	Lipid transporter that alters the lipid composition of the luminal plasma membrane of CNS ECs, thereby preventing caveolae formation and caveolae-mediated transcytosis	^39, 40^
NSF	*N*-ethylmaleimide sensitive fusion	Regulates disassembly of SNARE complexes and their recycling	^41^
SNAP23	Synaptosomal-associated protein 23	Component of the endothelial SNARE machinery Regulates endothelial exocytosis	^42, 43^
PACSIN2	Protein kinase C and casein kinase substrate in neurons 2	F-BAR domain protein that regulates membrane sculpting of caveolae Stabilizes caveolae at the membrane	^44, 45^
VAMP2, VAMP3 or VAMP8	Vesicle-associated membrane protein-2, -3 or -8	SNARE complex protein present on vesicles Mediates fusion of vesicles with target membrane	^46, 47^

**Figure 5:**
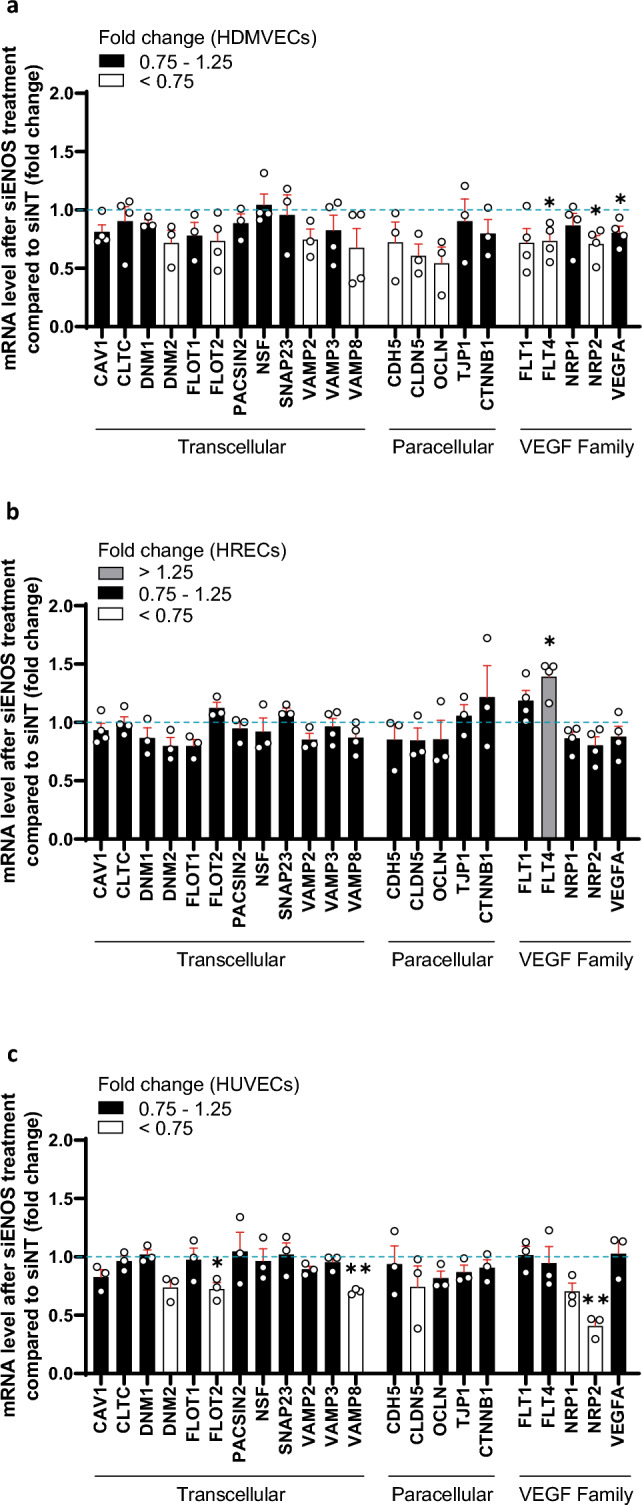
Silencing of *NOS3* expression has limited or no effects on the expression of various transcellular transport-related, paracellular transport-related and VEGF family-related transcripts in HDMVECs, HRECs and HUVECs. mRNA levels of various transcellular transport-related, paracellular transport-related and VEGF family-related transcripts in si*NOS3*-treated HDMVECs (**a**), HRECs (**b**) and HUVECs (**c**), in *n* = 3–4 independent experiments. Results are expressed as fold change relative to the siNT control. Data are represented as mean ± SEM. *p < 0.05; **p < 0.01 (one-sample t-test).

We found that silencing of *NOS3* expression altered the mRNA expression of only a few transcripts in HDMVECs, HRECs and HUVECs (Fig. 5a–c)*.* Silencing of *NOS3* expression decreased the expression of *FLOT2* and *VAMP8* in HUVECs (both by 1.4-fold), but not in HDMVECs and HRECs (Fig. 5a–c). In addition, silencing of *NOS3* expression reduced the expression of *NRP2* in HDMVECs and HUVECs (by 1.4-fold and 2.5-fold, respectively), but not in HRECs (Fig. 5a–c). Moreover, silencing of *NOS3* expression increased *FLT4* (also known as VEGFR3) mRNA levels in HRECs (by 1.4-fold), lowered *FLT4* expression in HDMVECs (by 1.4-fold) and had no effect on *FLT4* expression in HUVECs (Fig. 5a–c). Silencing of *NOS3* expression lowered *VEGFA* mRNA levels in HDMVECs (by 1.2-fold), but not in HRECs and HUVECs (Fig. 5a–c). An overview of the mRNA data presented in arbitrary units is shown in Supplementary Figs. S9–S11.

Retinal ECs express various specialized proteins such as MFSD2A that maintain the characteristic properties of blood-retinal barrier ECs such as low rates of caveolae-mediated transport^39, 40^. Therefore, we determined whether silencing of *NOS3* expression affects *MFSD2A* expression only in retinal ECs. Silencing of *NOS3* expression reduced *MFSD2A* mRNA levels in HRECs (by 1.4-fold; Supplementary Fig. S12a). In contrast, silencing of *NOS3* expression had no effect on *MFSD2A* expression in HRECs in the presence of exogenous VEGF-A (Supplementary Fig. S12a). Moreover, pre-treatment with the NOS inhibitor l-NAME did not alter *MFSD2A* expression both in the presence and absence of VEGF-A stimulation in HRECs and BRECs (Supplementary Fig. S12b).

Endothelin-1 (ET-1) and NO are generally considered to be each others counterpart, with ET-1 acting as a vasoconstrictor and NO as a vasodilator^48^. VEGF increases the expression and secretion of ET-1 in ECs^49^. ET-1, in turn, has been shown to regulate eNOS expression and activity^50, 51^, as well as endothelial permeability^52–55^. In our experiments, we found that silencing of *NOS3* expression did not significantly alter ET-1 mRNA (*EDN1*) expression in HDMVECs, HRECs and HUVECs. However, a consistent tendency towards lower *EDN1* expression was observed across all HDMVEC donors following the silencing of *NOS3* expression (Supplementary Fig. S13).

In conclusion, silencing of *NOS3* expression does not alter or only modestly affects the expression of most tested transcellular transport-related, paracellular transport-related and VEGF family-related genes. In addition, differential transcriptional responses of *NOS3* silencing were detected between the three human EC subtypes.

## Discussion

In the present study, we have shown that eNOS plays a differential role in the regulation of late effects of VEGF-A on endothelial hyperpermeability in various cultured EC phenotypes, underscoring the complexity of VEGF-A signal transduction pathways. In HDMVECs, eNOS partially regulated the effects of VEGF-A on fluid phase-dependent tracer permeability (70 kDa-dextran and 766 Da Cy3 tracers), but not in HRECs and HUVECs. On the other hand, VEGF-A-induced receptor-mediated transport of BSA^56, 57^ was not affected by eNOS in HDMVECs, HRECs and HUVECs. In addition, this study demonstrates that eNOS regulates basal permeability in HRECs.

To understand these differences in eNOS functions in the regulation of endothelial permeability, we focused on studying transcellular permeability, as we have previously shown that this pathway is activated several days after exposure to VEGF-A^20, 21^. Endocytosis is the first essential step of transcytosis, and on the basis of our tracer uptake experiments presented in this study, we were unable to draw firm conclusions whether tracer uptake is affected. On the basis of these experiments, at most, a tendency of increased intracellular localization of tracer after silencing of *NOS3* expression in HDMVECs and HRECs was observed. The increased intracellular localization of tracers may imply that silencing of *NOS3* expression either promotes tracer uptake, or inhibits tracer exocytosis, which requires further investigation.

We analyzed how silencing of *NOS3* expression regulates expression of endothelial permeability-related transcripts. The expression of most permeability-related transcripts was at most modestly affected by silencing of *NOS3* expression, with the exception of *PLVAP* in HDMVECs and HUVECs, and *KDR* and *NRP2* in HUVECs (which were ≥ two-fold differentially expressed).

PLVAP is a crucial regulator of protein transport in fenestrated endothelium^25^. However, the relevance of PLVAP in permeability of continuous endothelium likely varies between EC phenotypes. Several studies demonstrate that PLVAP expression is not relevant for basal permeability of continuous endothelium, with the exception of lung endothelium^21, 32, 58^. PLVAP is in most EC phenotypes probably only relevant in pathological endothelial hyperpermeability. For instance, silencing of *PLVAP* expression in cultured BRECs reduces VEGF-A-induced permeability, but not basal permeability^21^. In addition, VEGF- and histamine-induced permeability, but not basal permeability, is reduced in dermal endothelium of PLVAP heterozygous mice^59^. In line with these reports, we found in the present study that the expression levels of *PLVAP* were lower in HDMVECs and HUVECs when *NOS3* was silenced in the absence of VEGF-A stimulation, whereas basal permeability remained unaffected. Moreover, the expression of *PLVAP* was not altered by silencing of *NOS3* expression in HRECs, whereas basal permeability was increased. This suggests that the expression of PLVAP does regulate basal permeability in these situations. We also found that eNOS regulates VEGF-A-induced expression of *PLVAP* in HDMVECs and HUVECs, whereas VEGF-A-induced fluid-phase dependent permeability was only affected in HDMVECs by silencing of *NOS3* expression. These data suggest that PLVAP is not the primary contributor to VEGF-A-induced hyperpermeability in human cultured ECs, highlighting significant differences among endothelial phenotypes. This hypothesis is further supported by our PLVAP overexpression experiment in HDMVECs.

A notable observation in our present study is that silencing of *NOS3* expression reduced VEGFR2 mRNA and protein expression as well as *NRP2* mRNA expression in HUVECs, whereas VEGF-A-induced hyperpermeability remained unaffected. A large portion of VEGFR2 is localized intracellularly in endosomes (approximately 40%), and is not directly available for binding to VEGF-A^60–62^. It is therefore possible that silencing of *NOS3* expression affects only the endosomal pool of VEGFR2, and not the membrane-associated pool of VEGFR2. In addition, there appear to be notable differences in expression of various VEGF family-related transcripts between the different EC subtypes. For instance, the relative expression of *VEGFA* was higher in HUVECs compared to the other types of ECs, whereas the expression of *NRP1* and *NRP2* was lower. Additional research is needed to understand these findings in the context of our Transwell permeability data.

We were unable to identify the underlying mechanism explaining the differential responses of EC phenotypes. Whereas *NOS3* regulates PLVAP expression in HDMVECs and HUVECs, it seems unlikely that PLVAP plays a functional role in the differential permeability response. Notably, our focus was primarily on the transcellular pathway, which is often overlooked in studies of endothelial hyperpermeability mechanisms. Further investigation is needed to determine how silencing of *NOS3* expression affects the paracellular pathway. Preliminary experiments did not reveal any evidence that silencing of *NOS3* expression affects VEGF-induced paracellular gap formation (data not shown).

It is likely that some of the observed responses in si*NOS3*-treated cells can not simply be attributed to reduced NO availability due to the absence of eNOS. This is further illustrated when comparing the differential effects of si*NOS3* treatment versus NO donor SNAP treatment on *PLVAP* expression. This underscores the importance of investigating other reaction products of eNOS to gain a better understanding of the underlying mechanism. For instance, with regards to *PLVAP,* it can be hypothesized that eNOS-derived NO itself may have a positive regulatory effect on PLVAP expression, as suggested by the si*NOS3* data. However, when cells produce excessive amounts of NO and ONOO^−·^ is formed, ONOO^−·^ may downregulate PLVAP expression.

At present, it remains unclear what accounts for the varying importance of eNOS in endothelial permeability among different EC phenotypes. These cells differ in tissue of origin, vascular caliber and physiological characteristics, suggesting potential variations in post-translational modifications or protein–protein interactions between cell types. Since we observed an eNOS-dependent effect on permeability exclusively in HDMVECs, it is likely that an unidentified player in the VEGF/eNOS pathway is either expressed or functionally important specifically in HDMVECs. A detailed analysis of how silencing of *NOS3* expression affects gene expression with single cell sequencing or proteomics may contribute to a better understanding of the differential roles of eNOS in the late effects of VEGF-A on hyperpermeability. One of the regulators of eNOS activity in cells is CAV1, which negatively regulates eNOS activity through direct interaction^63, 64^. After stimulation with VEGF-A, CAV1 dissociates from eNOS in a hsp90-dependent manner and becomes activated^65, 66^. In our experiments, we observed a significant higher expression level of *CAV1* in HDMVECs compared to HRECs and HUVECs (Supplementary Figs. S9a, S14). Whereas we did not directly investigate eNOS activity in the present study, these findings with *CAV1* illustrate that protein–protein interactions may vary between cells types.

In conclusion, our results demonstrate that eNOS has varying functional involvement in the late effects of VEGF-A on endothelial hyperpermeability in different human endothelial phenotypes. VEGF-A induces delayed endothelial hyperpermeability via both eNOS-dependent and eNOS-independent mechanisms. Specifically, eNOS partially regulates VEGF-A-induced endothelial hyperpermeability in HDMVECs but not in HRECs and HUVECs. The underlying mechanism for these variations of eNOS involvement remains unidentified. Further investigation is needed to determine whether the differential late effects of eNOS in endothelial hyperpermeability are specific to the VEGF-A pathway or occur downstream of other permeability-inducing agents as well. In addition, our study provides further insight into the regulation of PLVAP expression in ECs, which appears to be dependent on eNOS function in HDMVECs and HUVECs.

## Materials and methods

### Cell cultures

For use of human cells and serum, approval by the Medical Ethical Review Committee of the Academic Medical Center Amsterdam was given. Subjects gave informed consent for the use of tissues or serum, and samples were stored anonymously. Human serum and umbilical cords were collected according to the principles of conduct for research integrity as described in the Research Code AMC VUmc, §3.2.

HDMVECs, a kind gift of dr. P. Koolwijk (Amsterdam UMC, location Free University, The Netherlands), were grown on 2% gelatin (Merck Millipore)-coated plastic tissue culture plates in 50% M199 medium supplemented with 10% heat-inactivated human serum (obtained from the Department of Oncology, Amsterdam UMC, location Academic Medical Center, The Netherlands), 10% fetal bovine serum (Gibco), 1% penicillin–streptomycin-glutamine (Gibco), and 50% EGM-2 MV medium (Lonza) supplemented with 1% penicillin–streptomycin (Gibco). HDMVECs were cultured at 37 °C and 5% CO_2_, and used for experiments at passage 6 to 12. Experiments were carried out with HDMVECs of at least 3 different donors, unless noted otherwise.

HRECs, purchased from Cell Systems (ACBRI 181), were grown in EGM-MV2 medium (Promocell) on 10 µg/mL fibronectin (Merck Millipore)-coated plastic plates. HRECS were cultured at 37 °C and 5% CO_2_, and used at passage 6 to 10.

HUVECs were isolated from umbilical cords (obtained from the Department of Gynecology, Amsterdam UMC, location AMC, Amsterdam, The Netherlands) as described previously^67^. HUVECs were grown in M199 medium (Gibco) supplemented with 10% heat-inactivated human serum, 10% fetal bovine serum (FBS) (Gibco), 1% penicillin–streptomycin-glutamine (Gibco) on 2% gelatin-coated plastic tissue culture plates during most experiments. During the Transwell permeability experiments, HUVECs were cultured in EGM2 medium (PromoCell). HUVECs were cultured at 37 °C in 5% CO_2_, and used at passage 4. Each experiment was carried out with HUVECs of at least 3 different donors.

BRECs were isolated from freshly enucleated cow eyes obtained from the slaughterhouse as described previously^68^. All blood vessels in the retina were isolated, which include arteries, arterioles, capillaries, venules and veins. BRECs were grown on collagen type IV (Sigma-Aldrich; 0.01 mg/mL in 0.01% v/v acetic acid)- and 10 µg/mL fibronectin-coated plastic plates in DMEM containing 25 mM HEPES and 4.5 g/L glucose (Lonza), supplemented with 10% fetal bovine serum, 1 × non-essential amino acids in minimum essential medium (Thermo Fisher Scientific), fungizone antimycotic (Gibco), 1% penicillin–streptomycin-glutamine, 2 mM l-glutamine (Thermo Fisher Scientific) and 10 μg/mL hydrocortisone (Sigma-Aldrich). BRECs were cultured at 37 °C and 10% CO_2_ and used at passage 1.

Cells were treated with recombinant human VEGF-A_165_ (Acro) or S-nitroso-N-acetylpenicillamine (SNAP; Cayman Chemical) as indicated in the Results section and in figure legends. Cells were cultured for 4 h or overnight in M199 medium supplemented with 2% heat-inactivated human serum and 1% penicillin–streptomycin before VEGF-A or SNAP treatment, respectively.

### siRNA knockdown

Gene silencing was achieved by treating cells with ON-TARGETplus siRNA (Dharmacon) according to the reverse transfection method described by the manufacturer. Briefly, cells were transfected with 25 nM of either the eNOS-specific smartpool mix (si*NOS3*; #L-006490-00) or the non-targeting control pool mix (siNT; #D-001810-10), and 2.5 µg/mL DharmaFECT 1 transfection reagent (Dharmacon) in M199 medium supplemented with 2% heat-inactivated human serum. To avoid cytotoxicity, transfection medium was replaced by complete medium at 6 h after siRNA transfection. Cells were lysed or fixed at 72 h after siRNA treatment, unless stated otherwise. For the permeability experiments, cells were used at 96 h post-transfection to enable cells to form a stable barrier before VEGF-A treatment.

### Tracer permeability experiments

Permeability experiments were performed as described previously with minor adjustments^62^. Cells were seeded on coated 24-well Transwell inserts (0.33 cm^2^, pore size 0.4 μm; Greiner Bio-One) to grow to confluence. Transwell inserts were coated with 2% gelatin (HDMVECs) or 10 µg/mL fibronectin (HDMVECs, HRECs, HUVECs). Permeability was determined at 48 h after VEGF-A stimulation. Cells were stimulated basolaterally with VEGF-A for 24 h, followed by a second 24 h incubation period in fresh medium. On the experimental day, fluorescent tracer molecules, 250 µg/mL FITC-conjugated bovine albumin (BSA-FITC, 67 kDa, Invitrogen, #A23015), 250 µg/mL of FITC-conjugated dextran (dextran-FITC, 70 kDa, Sigma, #46945), or 50 µg/mL Cy3-tracer (766 Da, GE Healthcare, #PA23001) were added to the apical side of the Transwell insert, and samples were collected from the basolateral compartment after 2 h. Fluorescence intensity of samples was measured using a microplate reader. The intensity of fluorescence in the basolateral compartment samples was considered to be a measure for permeability and is presented relative to that of the non-targeting siRNA control^62^.

### Dextran or BSA internalization assays

Dextran internalization experiments were performed as described previously with minor adjustments^69^. Briefly, cells were grown to confluence on 10 µg/mL fibronectin-coated glass coverslips. Next, cells were incubated with 250 µg/mL of 70 kDa dextran-Texas Red (lysine fixable, Invitrogen, #D1864) or 100 µg/mL of BSA-FITC for 10 min at 37 °C, followed by two washes with Hank's balanced salt solution. Cells were then fixed for 20 min in 4% paraformaldehyde (Electron Microscopy Sciences) at room temp followed by treatment with 0.15% glycine in Hank's balanced salt solution for at least 15 min at room temp. Coverslips were mounted using a mounting medium containing 4′,6-diamidino-2-phenylindole (DAPI) (Vectashield, Vector Laboratories). Cells were imaged using a confocal laser scanning microscope (Leica, SP8, 63 × objective) at a z-stack interval of 0.3 µm with settings kept constant between conditions. The series of images through the z-plane were processed to form a 2D projected image. At least 5 different areas were imaged per condition per experiment. All images of the same experiment were equally adjusted for contrast. The amount of internalized dextran was calculated by quantifying the integrated density of the dextran signal using ImageJ software. This value was normalized to the total number of nuclei observed in the frame, and then subtracted by the normalized integrated density of the dextran signal in images where dextran was not added.

### Immunofluorescence

Cells grown on 10 µg/mL fibronectin-coated glass coverslips were fixed in 4% paraformaldehyde for 15 min at room temp, and washed 3 times with Hank's balanced salt solution. Coverslips were incubated in 10% normal donkey serum or 10% normal goat serum depending on the secondary antibody used and 0.1% Triton-X-100 for 1 h at room temp to block non-specific background staining. Next, coverslips were incubated with anti-PLVAP antibody (Leiden Universitair Medisch Centrum, clone PAL-E^26^, 1:50) or CAV1 antibody (BD BioScience, #610060, 1:500) diluted in normal antibody diluent (ScyTek Laboratories, #APG500) for 2 h at room temp. Primary antibodies were omitted for negative controls. After 3 washes with Hank’s balanced salt solution, coverslips were incubated with secondary antibodies (Jackson ImmunoResearch or Invitrogen) for 1 h at room temp. Coverslips were washed again 3 times with Hank’s balanced salt solution and mounted in Vectashield mounting medium containing DAPI. Cells on the coverslips were imaged and analyzed as described in the previous section.

### RNA isolation and mRNA quantification

Total RNA was isolated using TRIzol reagent (Invitrogen) according to the manufacturer’s instructions. An amount of 1 µg RNA was treated with DNase-I (amplification grade; Invitrogen) and reverse transcribed into cDNA using the Maxima First Strand cDNA Synthesis Kit (Thermo Scientific). Real-time quantitative PCR (RT-qPCR) was performed on 20 × diluted cDNA samples using a CFX96 real-time PCR detection system (Bio-Rad Laboratories). Specificity of the primers was confirmed as described previously^20^. Primer details are described in Table S1. Relative gene expression was calculated with LinRegPCR^70^. Data was normalized to the mean of 2 reference genes (tyrosine 3-monooxygenase/tryptophan 5-monooxygenase activation protein zeta (*YWHAZ*) and hydroxymethylbilane synthase (*HMBS*)) unless stated otherwise. The relative gene expression values represent arbitrary values, and were multiplied by 1000 for clarity reasons. To account for variability between donors/experiments, the data were normalized by dividing each individual data point by the mean of the corresponding control obtained in the same experiment. The resulting value was then multiplied by the mean value of all experiments. It is worth noting that we could not apply this normalization correction to *NOS1*, as *NOS1* was undetected in all HDMVECs controls.

### Western blotting

Western blotting was performed as described previously^62^. Cells were lysed in radioimmunoprecipitation assay buffer (Thermo Fisher Scientific) supplemented with protease inhibitor cocktail (Roche). Equal amounts of proteins were denatured in SDS-PAGE sample buffer, resolved by SDS-PAGE, and blotted onto nitrocellulose membranes. Membranes were blocked with 1:1 TBS:Intercept blocking buffer (LI-COR). Incubation with primary antibodies was performed overnight at 4 °C, followed by incubation with secondary antibodies for 1 h at room temp. eNOS was detected with an antibody from Cell Signaling Technology (#5880, 1:1000), VEGFR2 was detected with an antibody from Cell Signaling Technology (#2479, 1:1000), and β-actin was detected with an antibody from Sigma-Aldrich (#A5441, 1:5000). Proteins were visualized using the Odyssey system (LI-COR). Protein bands were analyzed by densitometric analysis using ImageJ.

### PLVAP overexpression

The expression of PLVAP was rescued in siNT- and si*NOS3*-treated HDMVECs using lentiviral transduction with the pReceiver-LV105 expression vector from GeneCopoeia (PLVAP-Lv105 vector). An empty Lv105 vector served as a control. Lentiviral particles were prepared by co-transfecting the construct with packaging vectors (pMD2G, RRE, pRSV/REV) into HEK293T cells. The day after siRNA transfection, HDMVECs were transduced with lentiviral particles, followed by a second transduction the next day with fresh lentiviral particles. During transduction, 5 µg/mL of Polybrene Infection/Transfection Reagent (Sigma-Aldrich, #TR-1003-G) was used to enhance the efficiency of transduction.

### Statistics

Data are represented as mean ± standard error of the mean (SEM). Two group comparisons were analyzed using an unpaired, two-tailed, Student’s *t*-test (parametric test) or a one-sample t-test (when comparisons were made to a normalized control). Statistical analyses and graph design were performed using GraphPad Prism 9. p values < 0.05 were considered to indicate significant differences, with levels of significance as follows: *p < 0.05; **p < 0.01; ***p < 0.001.

### Supplementary Information

Supplementary Information.

## Data Availability

The raw datasets generated during and/or analyzed during the current study will be made available upon request to the corresponding author.
