# Supplementary material for: Differential roles of eNOS in late effects of VEGF-A on hyperpermeability in different types of endothelial cells
Source: Sci Rep. 2023 Dec 5;13:21436. doi: 10.1038/s41598-023-46893-4 (PMC10698188; doi:10.1038/s41598-023-46893-4)
Supplement: Supplementary file 1 — Supplementary Information. [file 41598_2023_46893_MOESM1_ESM.docx]

**Differential roles of eNOS in late effects of VEGF-A on hyperpermeability in different types of endothelial cells**

Esmeralda K. Bosma ^1,2,3^, Shahan Darwesh ^1,2,3^, Yasmin I. Habani ^1,2,3^, Maxime Cammeraat ^1,2,3^, Paola Serrano Martinez ^1,2,3^, Mathilda E. van Breest Smallenburg ^1,2,3^, Jia Y. Zheng ^1,2,3^, Ilse M.C. Vogels ^1,2,3^, Cornelis J.F. van Noorden ^1,4^, Reinier O. Schlingemann ^1,2,3,5^ and Ingeborg Klaassen ^1,2,3^*.

^1^ Ocular Angiogenesis Group, Department of Ophthalmology, Amsterdam UMC location University of Amsterdam, Meibergdreef 9, Amsterdam, The Netherlands.
^2^ Amsterdam Cardiovascular Sciences, Microcirculation, Amsterdam, The Netherlands.
^3^ Amsterdam Neuroscience, Cellular & Molecular Mechanisms, Amsterdam, The Netherlands.
^4^ Department of Genetic Toxicology and Cancer Biology, National Institute of Biology, Ljubljana, Slovenia.
^5^ Department of Ophthalmology, University of Lausanne, Jules Gonin Eye Hospital, Fondation Asile Des Aveugles, Lausanne, Switzerland.

* Correspondence: [i.klaassen@amsterdamumc.nl](mailto:i.klaassen@amsterdamumc.nl)

**Supplementary Figures**


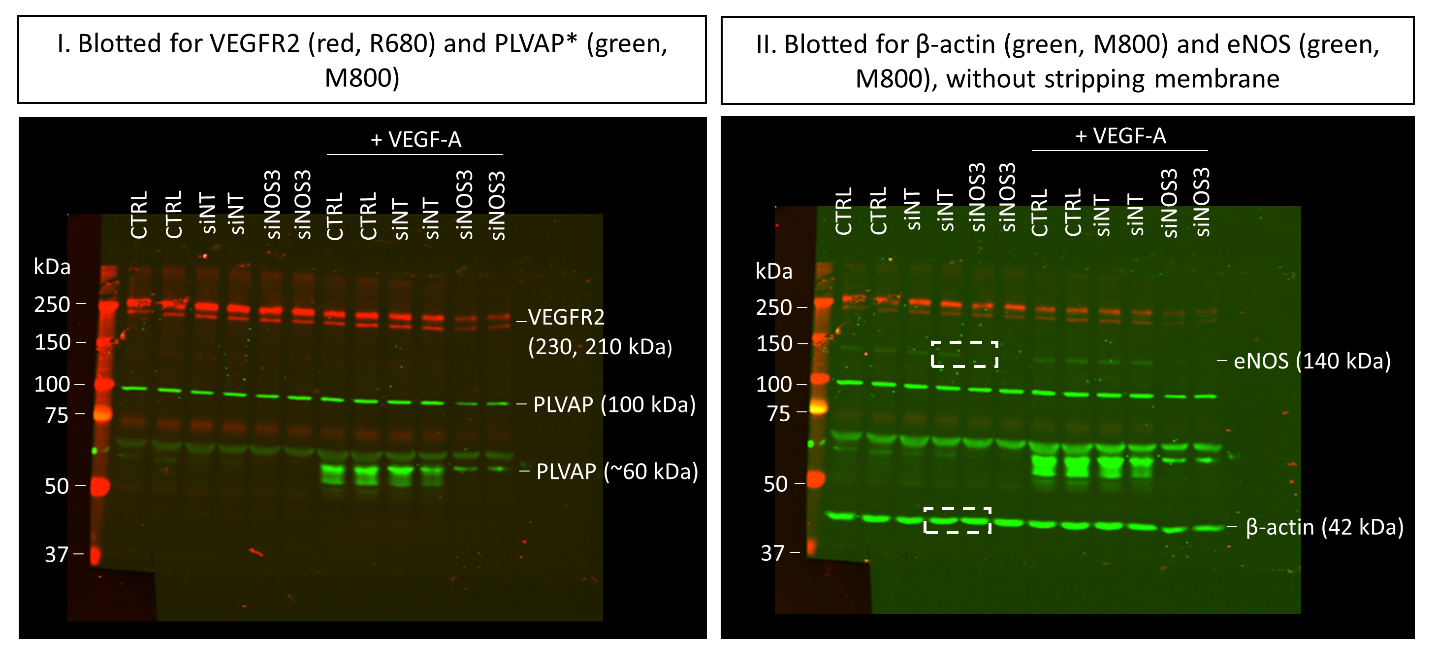


**Supplementary Fig. S1. Original blots for Fig. 1b (HDMVECs part).** Original blots of cropped western blot images of Fig. 1b (HDMVECs part). Cells were not transfected (CTRL) or transfected with siNT or si*NOS3*, and stimulated with VEGF-A for 48 h or left unstimulated. (a) First, the membrane was incubated with rabbit anti-VEGFR2 and mouse anti-PLVAP (abcam, #ab81719, clone 174/2) antibodies, and protein bands were visualized with IRDye680 anti-rabbit (red) and IRDye800 anti-mouse (green) antibodies. (b) Membranes were incubated with mouse anti-eNOS and mouse anti-β-actin antibodies, without stripping of the membrane. Protein bands were visualized with IRDye800 anti-mouse (green) antibody. Parts within the dashed boxes are shown in Fig. 1b.


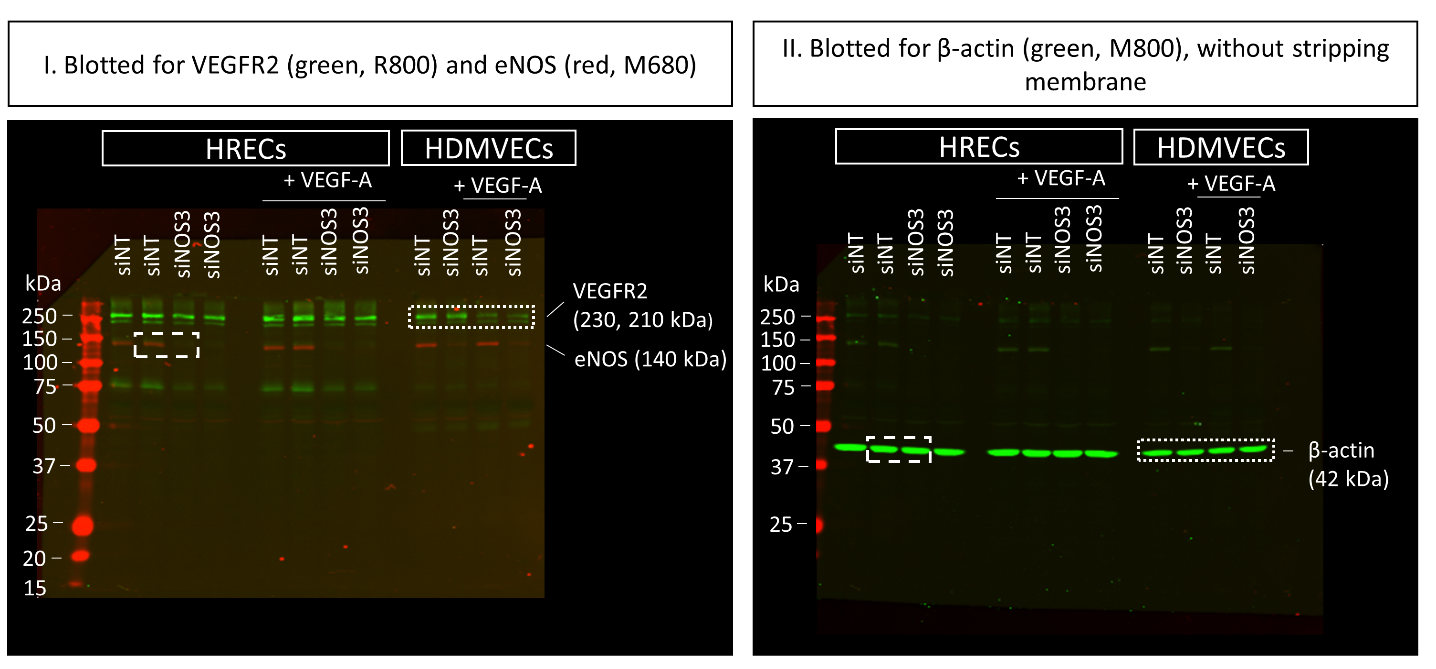


**Supplementary Fig. S2. Original blots for Fig. 1b (HRECs part) and Fig. 4b (HDMVECs part).**Original blots of cropped western blot images of Fig. 1b (HRECs part) and Fig. 4b (HDMVECs part). Cells were transfected with siNT or si*NOS3*, and stimulated with VEGF-A for 48 h or left unstimulated. (a) Firstly, the membrane was incubated with rabbit anti-VEGFR2 and mouse anti-eNOS antibodies, and protein bands were visualized with IRDye680 anti-mouse (green) and IRDye800 anti-rabbit (red) antibodies. (b) Secondly, the membrane was incubated with mouse anti-β-actin antibody, without stripping of the membrane. Protein bands were visualized with IRDye800 anti-mouse (green) antibody. Parts within the dashed boxes are shown in Fig. 1b, and parts within the dotted boxes are shown in Fig. 4b.


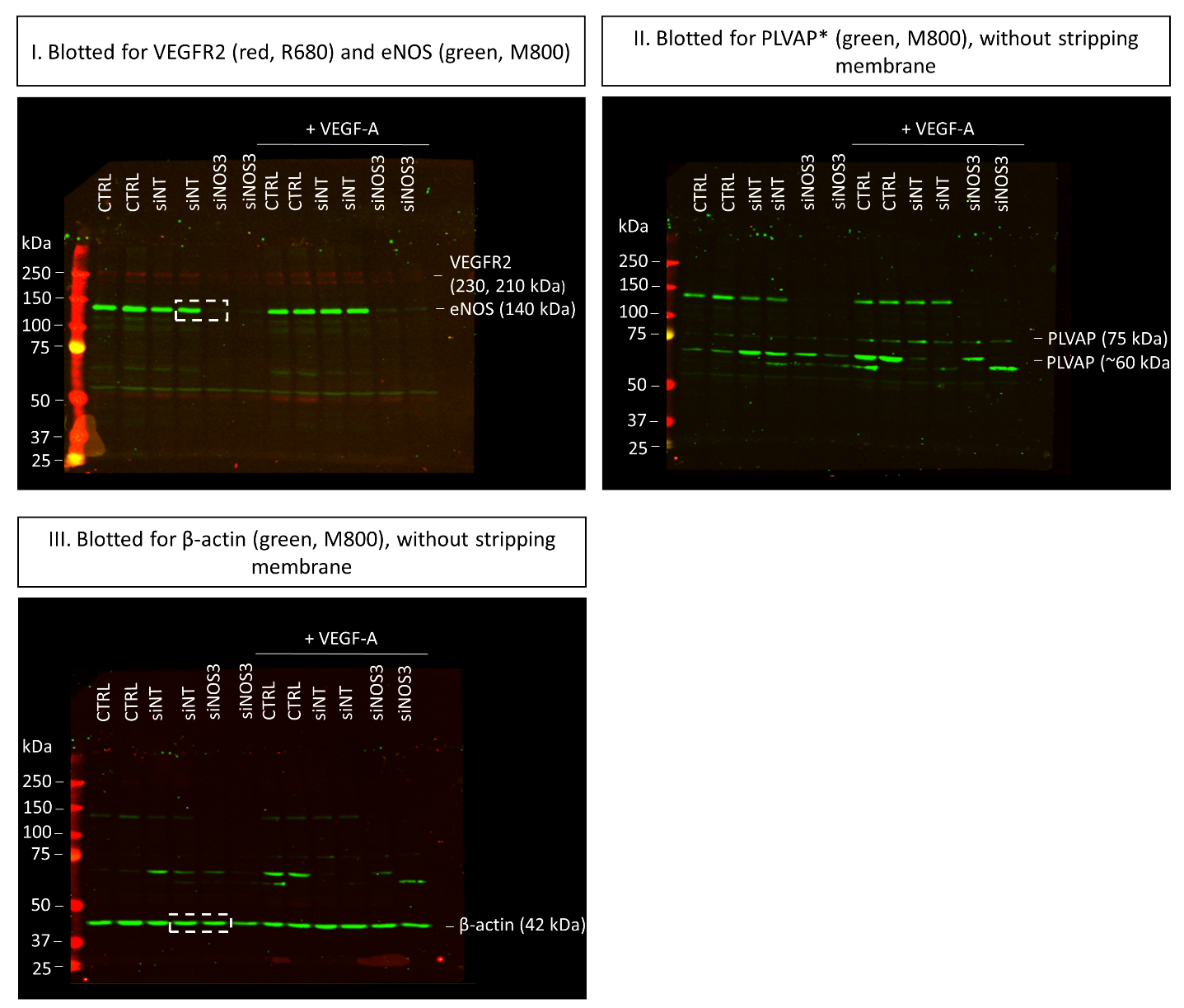


**Supplementary Fig. S3. Original blots for Fig. 1b (HUVECs part).**Original blots of cropped western blot images of Fig. 1b (HUVECs part). Cells were not transfected (CTRL) or transfected with siNT or si*NOS3*, and stimulated with VEGF-A for 48 h or left unstimulated. **(a)** Firstly, the membrane was incubated with rabbit anti-VEGFR2 and mouse anti-eNOS antibodies, and protein bands were visualized with IRDye680 anti-rabbit (red) and IRDye800 anti-mouse (green) antibodies. **(b)** Secondly, the membrane was incubated with mouse anti-PLVAP (abcam, #ab81719, clone 174/2), without stripping of the membrane, and protein bands were visualized with IRDye800 anti-mouse (green) antibody. **(c)** Thirdly, the membrane was incubated with mouse anti-β-actin antibody, without stripping of the membrane. Protein bands were visualized with IRDye800 anti-mouse (green) antibody. Parts within the dashed boxes are shown in Fig. 1b.

**
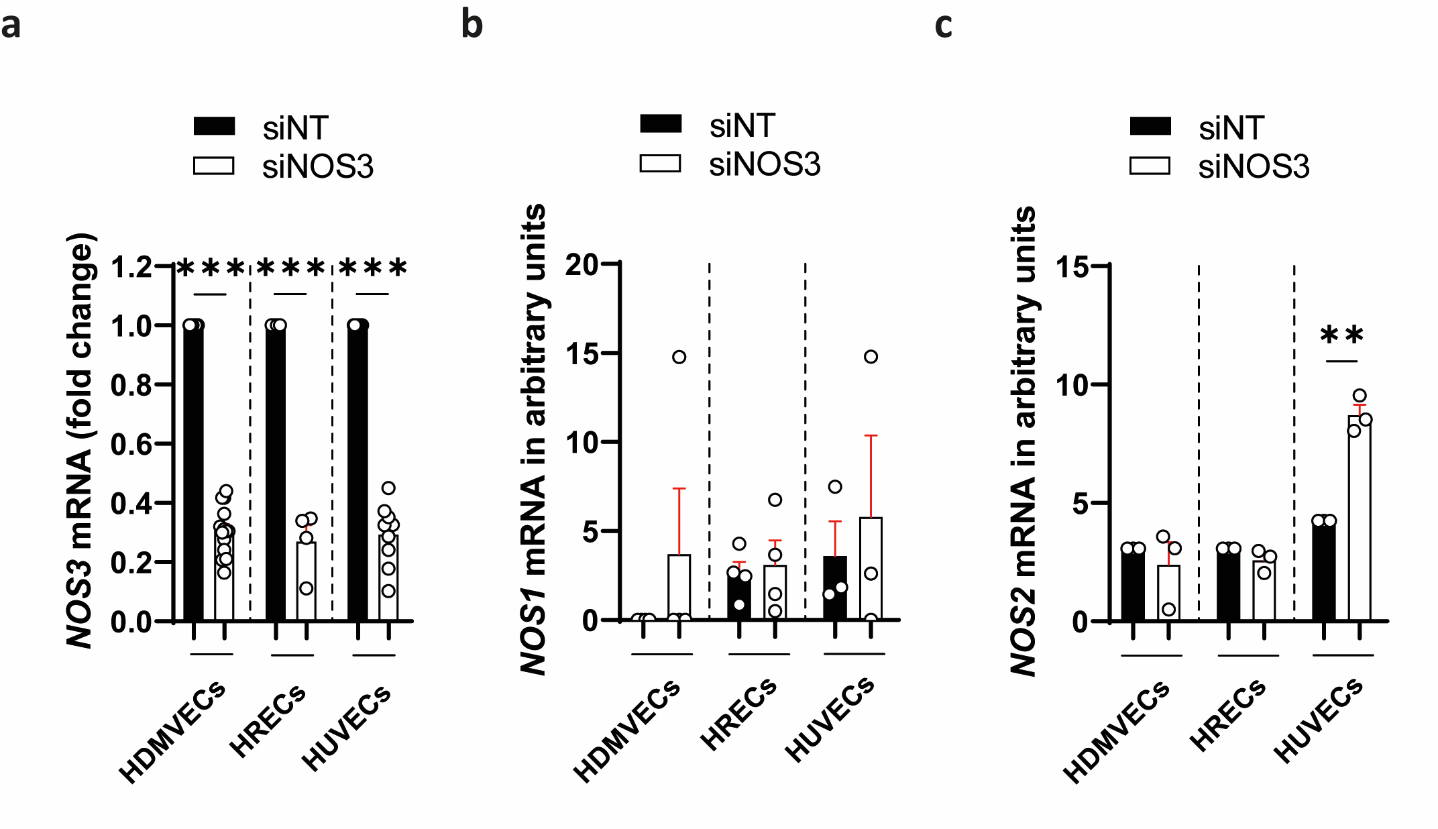
Supplementary Fig. S4. *NOS1-3* mRNA levels after silencing of *NOS3* expression.
(a)** Relative *NOS3* mRNA levels in control and si*NOS3*-treated HDMVECs, HRECs and HUVECs at 96 h after transfection, in *n* = 4-14 independent experiments. mRNA expression data were normalized to the expression of *YWHAZ* only, and are represented as fold change compared to the siNT-treated controls. **(b-c)** Relative *NOS1* **(b)** and *NOS2* **(c)** mRNA levels in control and si*NOS3*-treated HDMVECs, HRECs and HUVECs at 72 h after transfection, in *n* = 3-4 independent experiments. ∗∗p < 0.01, ∗∗∗p < 0.001; (one-sample t-test).

**
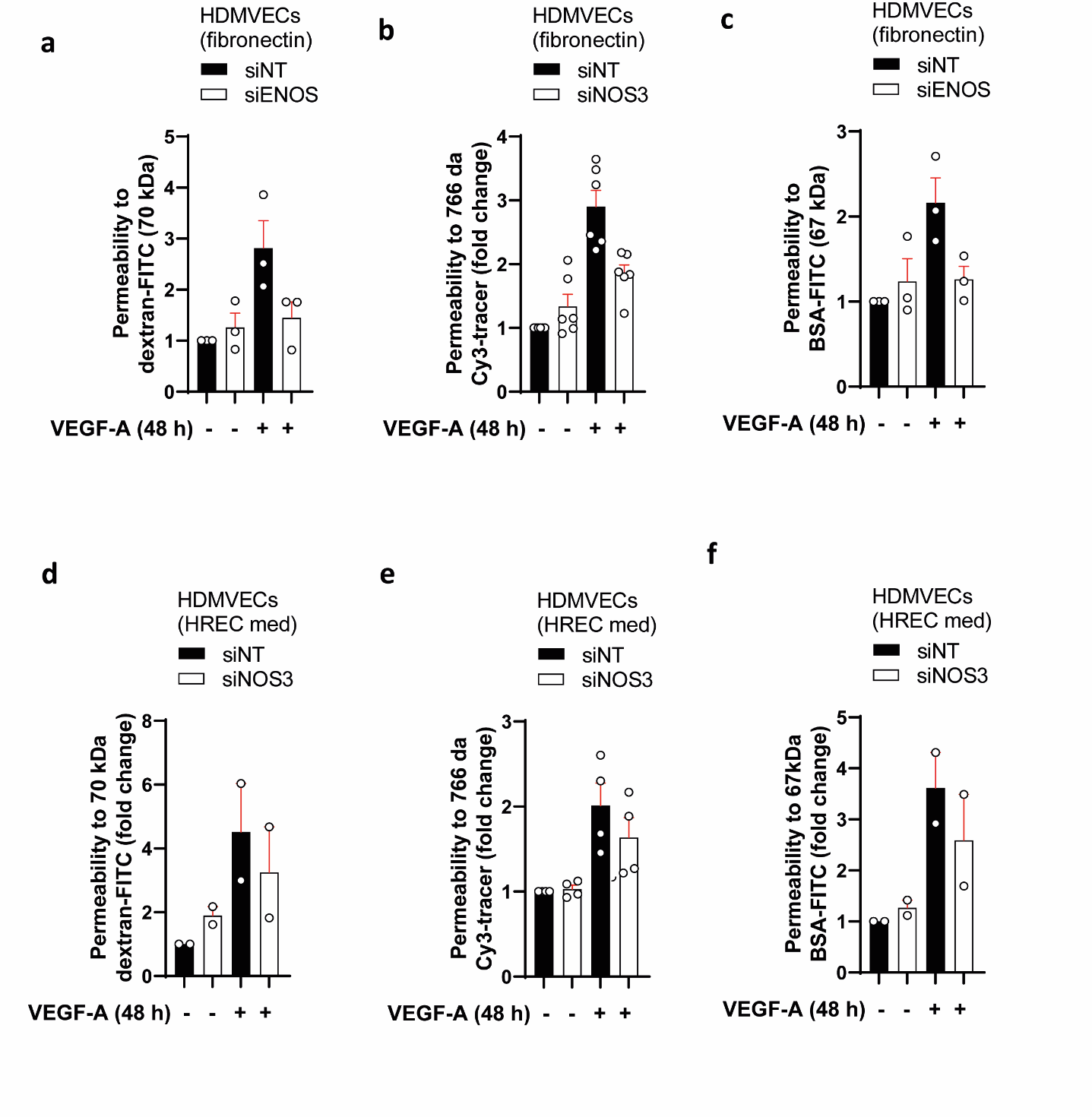
Supplementary Fig. S5. eNOS regulates VEGF-A-induced permeability in HDMVECs grown on fibronectin-coated Transwell inserts or cultured in HREC medium.**Permeability for dextran-FITC **(a,d)**, Cy3-tracer **(b,e)**, and BSA-FITC **(c,f)** in siNT- and si*NOS3*-treated HDMVECs cultured on fibronectin-coated Transwell inserts **(a-c)** or in HREC medium **(d-f)**. Cells were stimulated basolaterally with 25 ng/ml VEGF-A for 48 h. *n*= 2-6 independent experiments. Data are represented as mean ± SEM.**
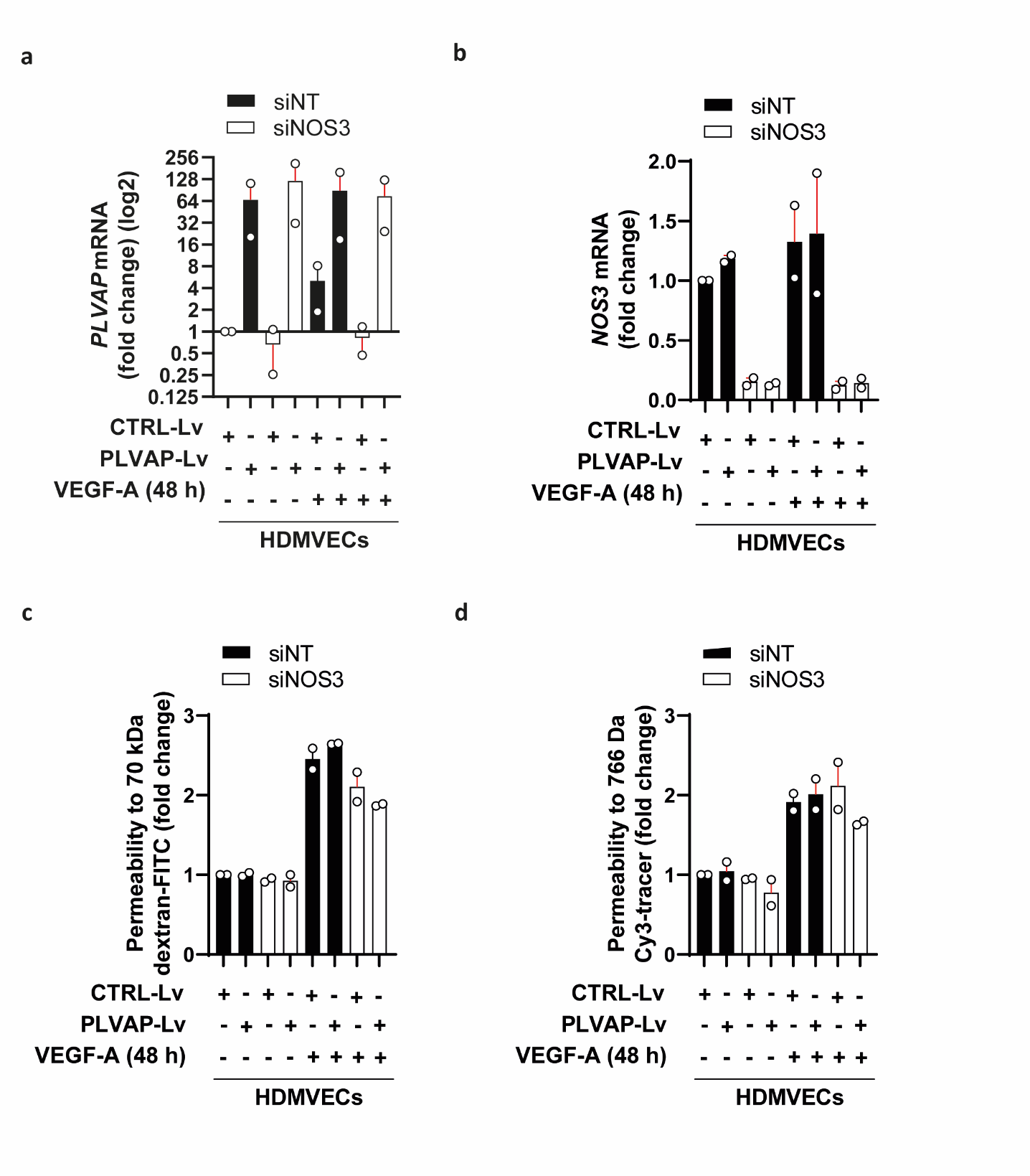
Supplementary Fig. S6. Overexpression of *PLVAP* does not alter tracer permeability in HDMVECs.**The expression of *PLVAP* was rescued in HDMVECs using the PLVAP-Lv105 expression vector (PLVAP-Lv), and subsequently a Transwell permeability experiment was performed. An empty Lv105 expression vector (CTRL-Lv) served as a control. **(a-b)** Relative *PLVAP* **(a)** and *NOS3* **(b)** mRNA levels in HDMVECs treated with siNT- or si*NOS3*-treated and CTRL-Lv or PLVAP-Lv at 96 h after siRNA transfection, in *n* = 2 independent experiments. Cells were stimulated with 25 ng/ml VEGF-A for 48 h. **(c-d)** Permeability for dextran-FITC **(c)** and Cy3-tracer **(d)** in HDMVECs treated with siNT or si*NOS3* and CTRL-Lv or PLVAP-Lv. In the permeability experiments, cells were stimulated basolaterally with 25 ng/ml VEGF-A for 48 h. *n* = 2 independent experiments. Data are represented as mean ± SEM.


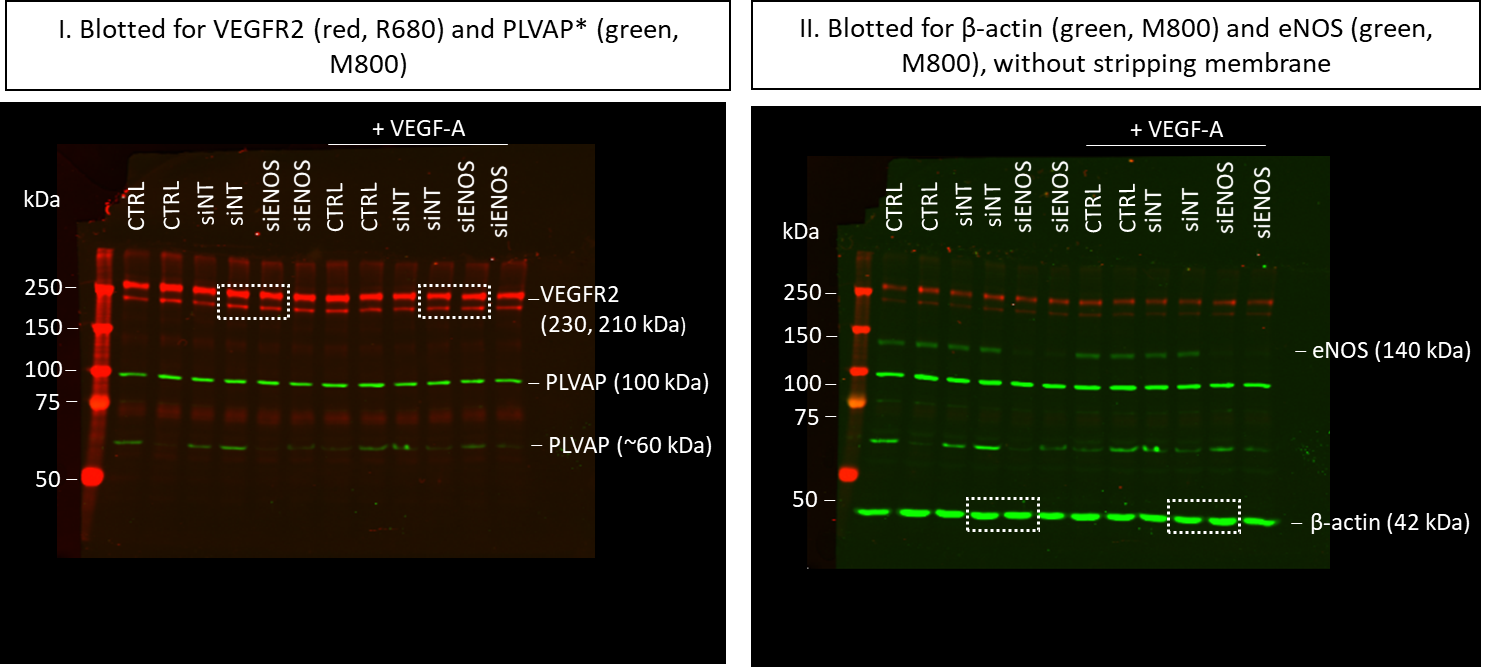


**Supplementary Fig. S7. Original blots for Fig. 4b (HRECs part).**Original blots of cropped western blot images of Fig. 4b (HRECs part). Cells were not transfected (CTRL) or transfected with siNT or si*NOS3*, and stimulated with VEGF-A for 48 h or left unstimulated. **(a)** Firstly, the membrane was incubated with rabbit anti-VEGFR2 and mouse anti-PLVAP (abcam, #ab81719, clone 174/2) antibodies, and protein bands were visualized with IRDye680 anti-rabbit (red) and IRDye800 anti-mouse (green) antibodies. **(b)** Secondly, the membrane was incubated with mouse anti-eNOS and mouse anti-β-actin antibodies, without stripping of the membrane. Protein bands were visualized with IRDye800 anti-mouse (green) antibody. Parts within the dotted boxes are shown in Fig. 4b.

**
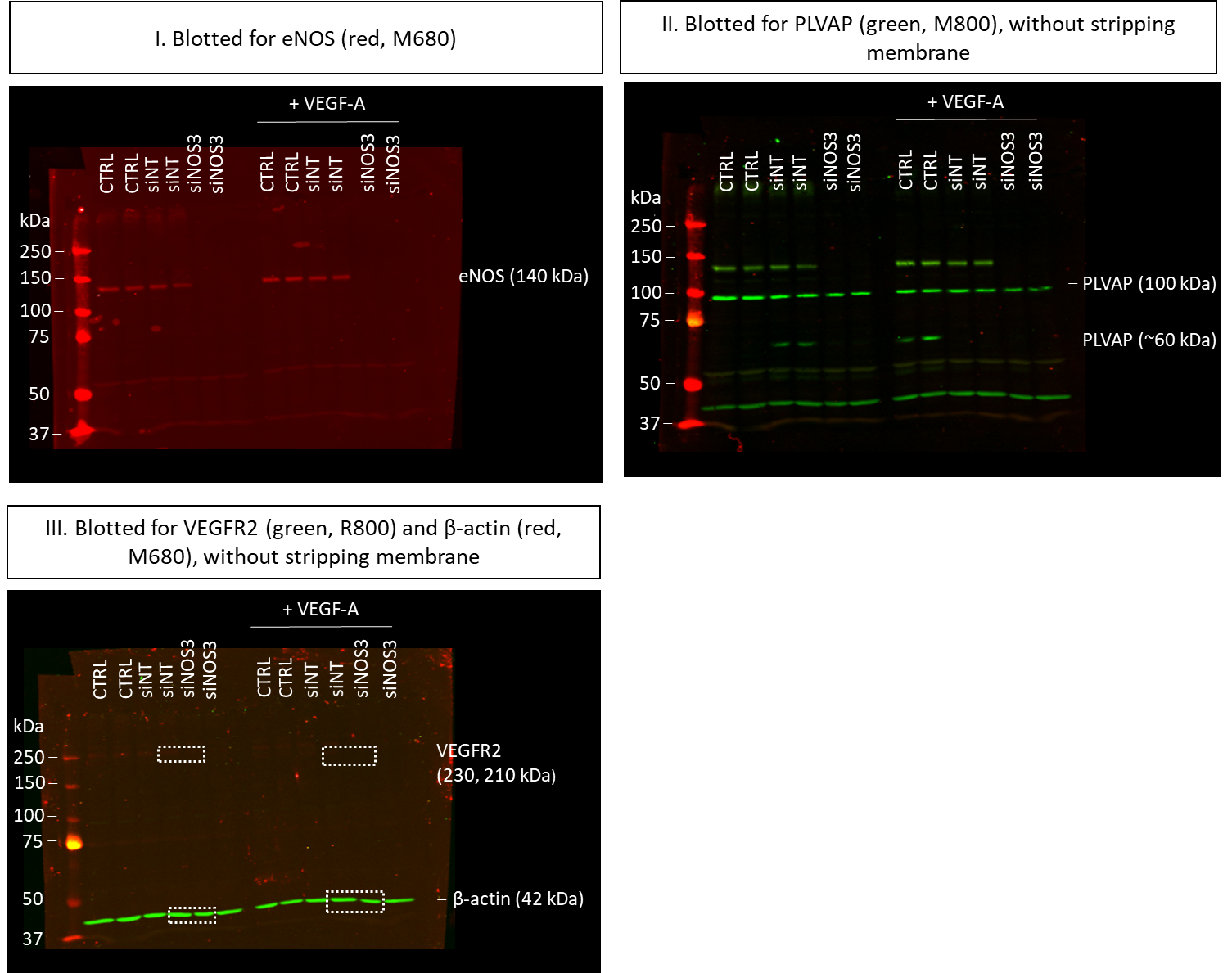
**

**Supplementary Fig. S8. Original blots for Fig. 4b (HUVECs part).**
Original blots of cropped western blot images of Fig. 4b (HUVECs part). Cells were not transfected (CTRL) or transfected with siNT or si*NOS3*, and stimulated with VEGF-A for 48 h or left unstimulated. **(a)** Firstly, the membrane was incubated with mouse anti-eNOS antibody, and protein bands were visualized with IRDye680 anti-mouse (red) antibody. **(b)** Secondly, the membrane was incubated with mouse anti-PLVAP (abcam, #ab81719, clone 174/2), without stripping of the membrane. Protein bands were visualized with IRDye800 anti-mouse (green) antibody. **(c)** Thirdly, the membrane was incubated with rabbit anti-VEGFR2 and mouse anti-β-actin antibodies, without stripping of the membrane. Protein bands were visualized with IRDye680 anti-rabbit (red) and IRDye800 anti-mouse (green) antibodies. Parts within the dotted boxes are shown in Fig. 4b.

**
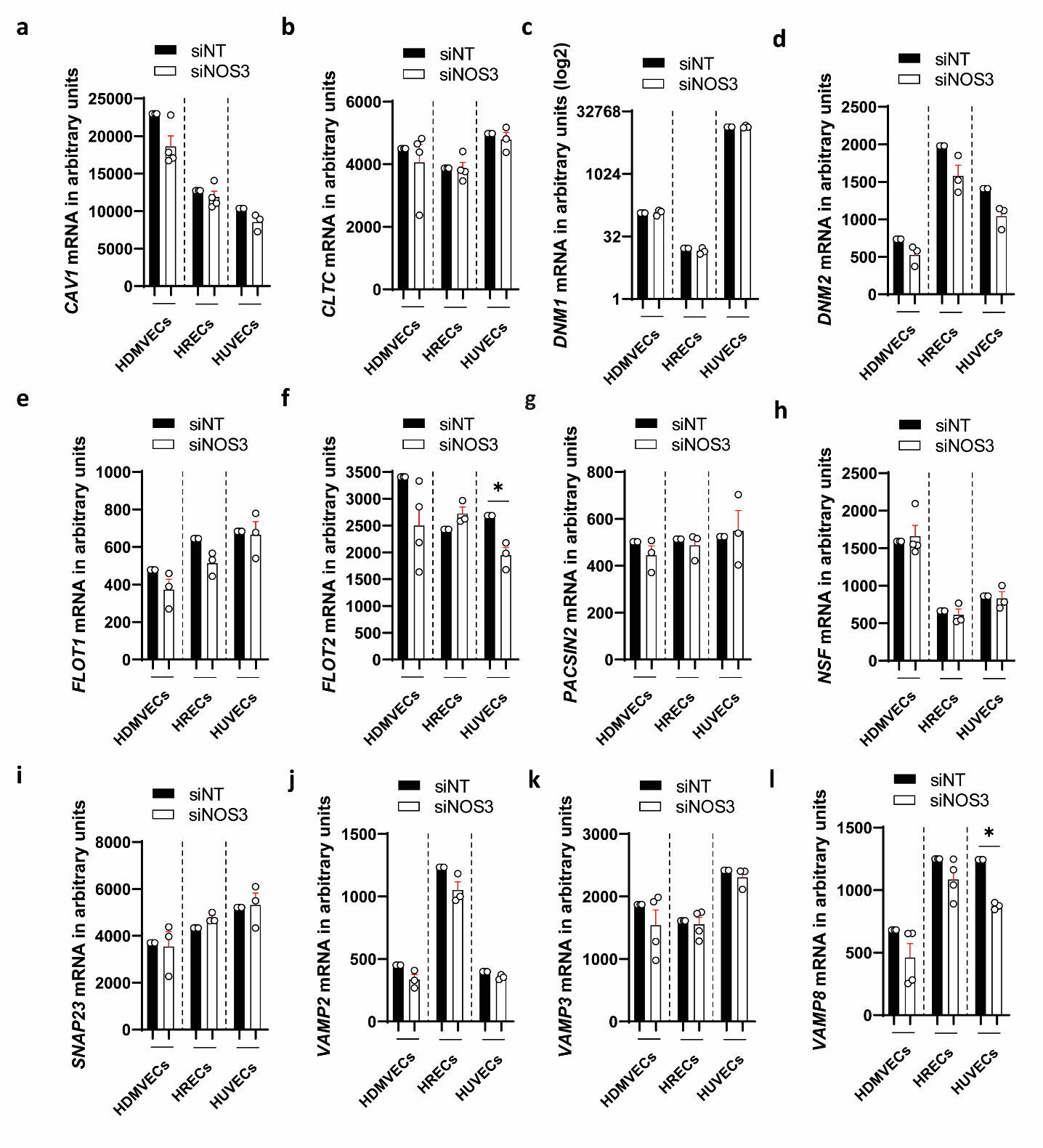
**

**Supplementary Fig. S9. Effect of *NOS3* silencing on the expression of transcellular transport-related transcripts in HDMVECs, HRECs and HUVECs.**mRNA data expressed in arbitrary units of transcellular transport-related genes in siNT- and si*NOS3*-treated HDMVECs, HRECs and HUVECs in *n* = 3-4 independent experiments. Results are normalized to the unstimulated siNT control. Data are represented as mean ± SEM. ∗p < 0.05 (one-sample t-test).

**
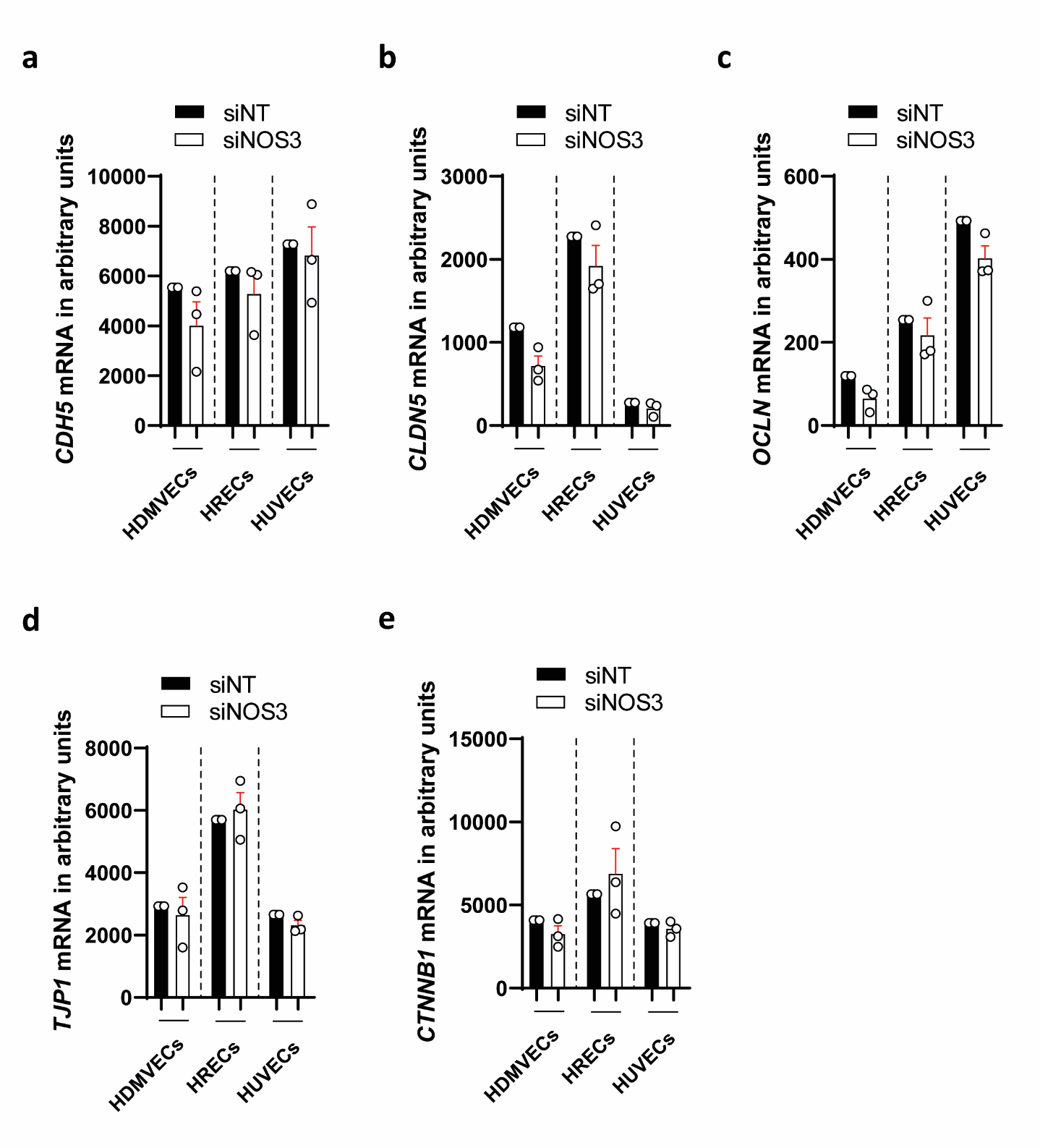
**

**Supplementary Fig. S10. Effect of *NOS3* silencing on the expression of paracellular transport-related transcripts in HDMVECs, HRECs and HUVECs.**mRNA data expressed in arbitrary units of paracellular transport-related genes in siNT- and si*NOS3*-treated HDMVECs, HRECs and HUVECs in *n* = 3-4 independent experiments. Results are normalized to the unstimulated siNT control. Data are represented as mean ± SEM.

**
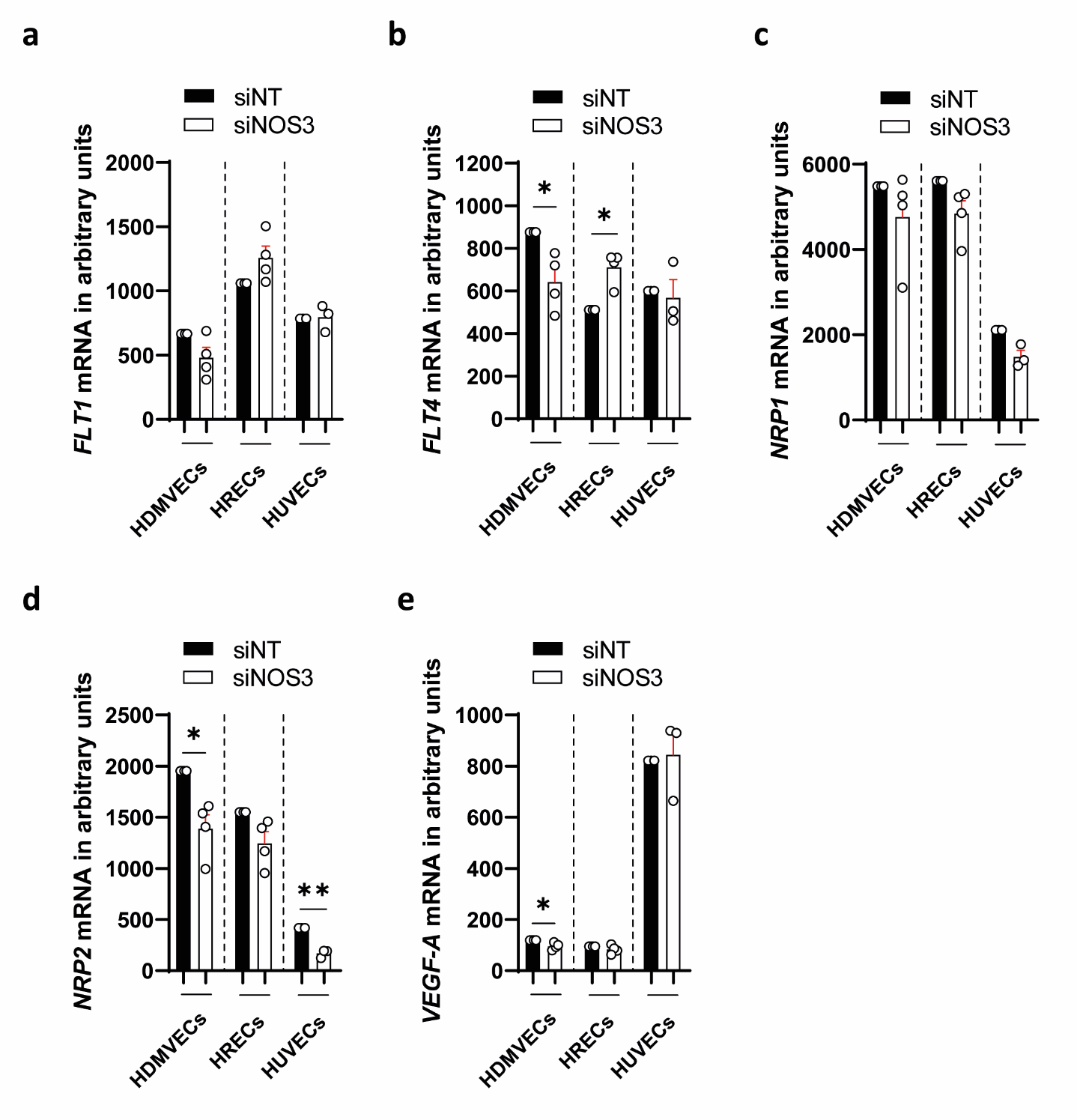
**

**Supplementary Fig. S11. Effect of *NOS3* silencing on the expression of VEGF family-related transcripts in HDMVECs, HRECs and HUVECs.**mRNA data expressed in arbitrary units of VEGF family-related genes in siNT- and si*NOS3*-treated HDMVECs, HRECs and HUVECs in *n* = 3-4 independent experiments. Results are normalized to the unstimulated siNT control. Data are represented as mean ± SEM. ∗p < 0.05; ∗∗p < 0.01 (one-sample t-test).

**
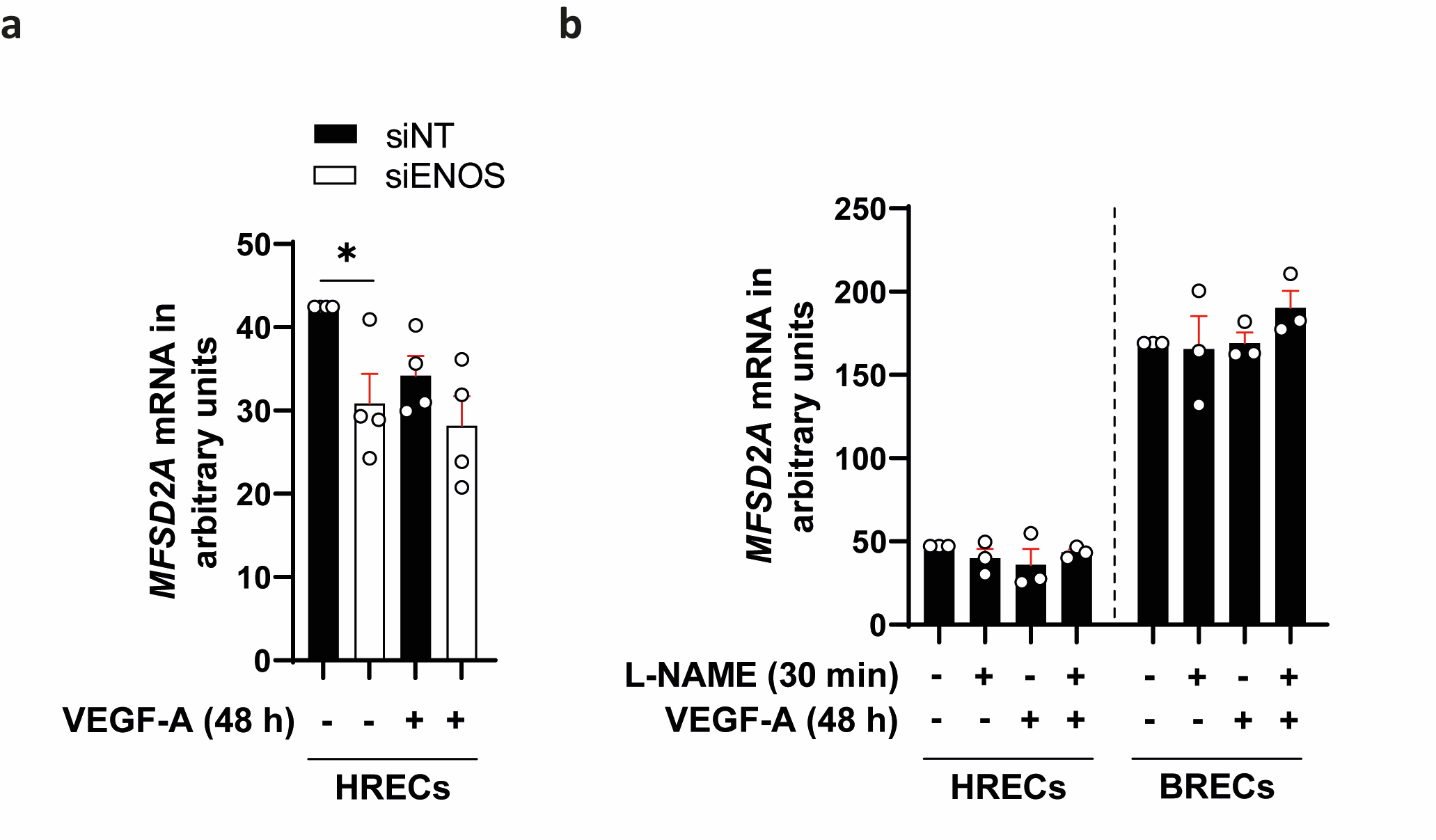
**

**Supplementary Fig. S12. Effect of silencing of eNOS expression and activity on *MFSD2A* expression in HRECs.
(a)** *MFSD2A* mRNA expression in arbitrary units in siNT- and si*NOS3*-treated HRECs in *n* = 4 independent experiments. Results are normalized to the unstimulated siNT control. **(b)** *MFSD2A* mRNA levels in control, L-NAME pretreated (100 µM, 30 min), VEGF-A-stimulated (25 ng/ml for 48 h) or a combination of L-NAME pretreated and VEGF-A-stimulated HRECs and BRECs, in *n* = 3 independent experiments. Results are normalized to the untreated control. Data are represented as mean ± SEM. ∗p < 0.05 (one-sample t-test or Student’s t-test).

**
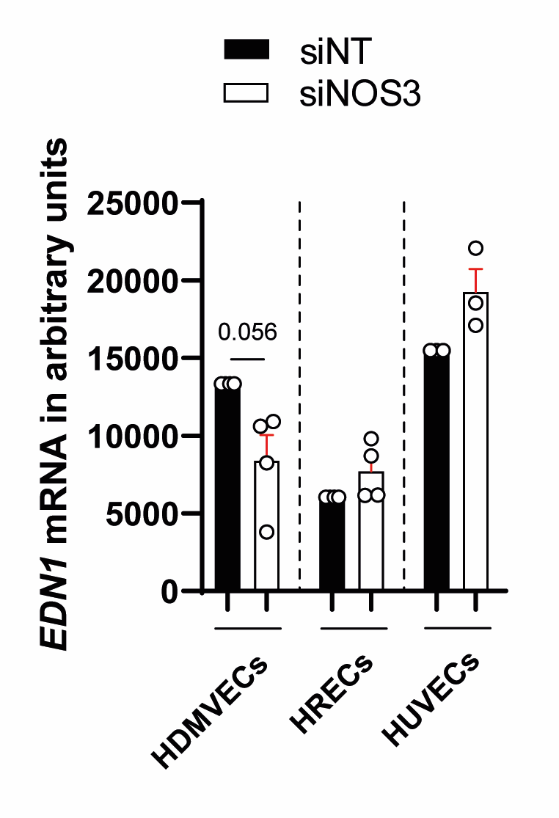
**

**Supplementary Fig. S13. Effect of silencing of *NOS3* expression on *EDN1* expression in HDMVECs, HRECs and HUVECs.**Relative *EDN1* mRNA levels in control and si*NOS3*-treated HDMVECs, HRECs and HUVECs at 72 h after transfection, in *n* = 3-4 independent experiments. (one-sample t-test).

**
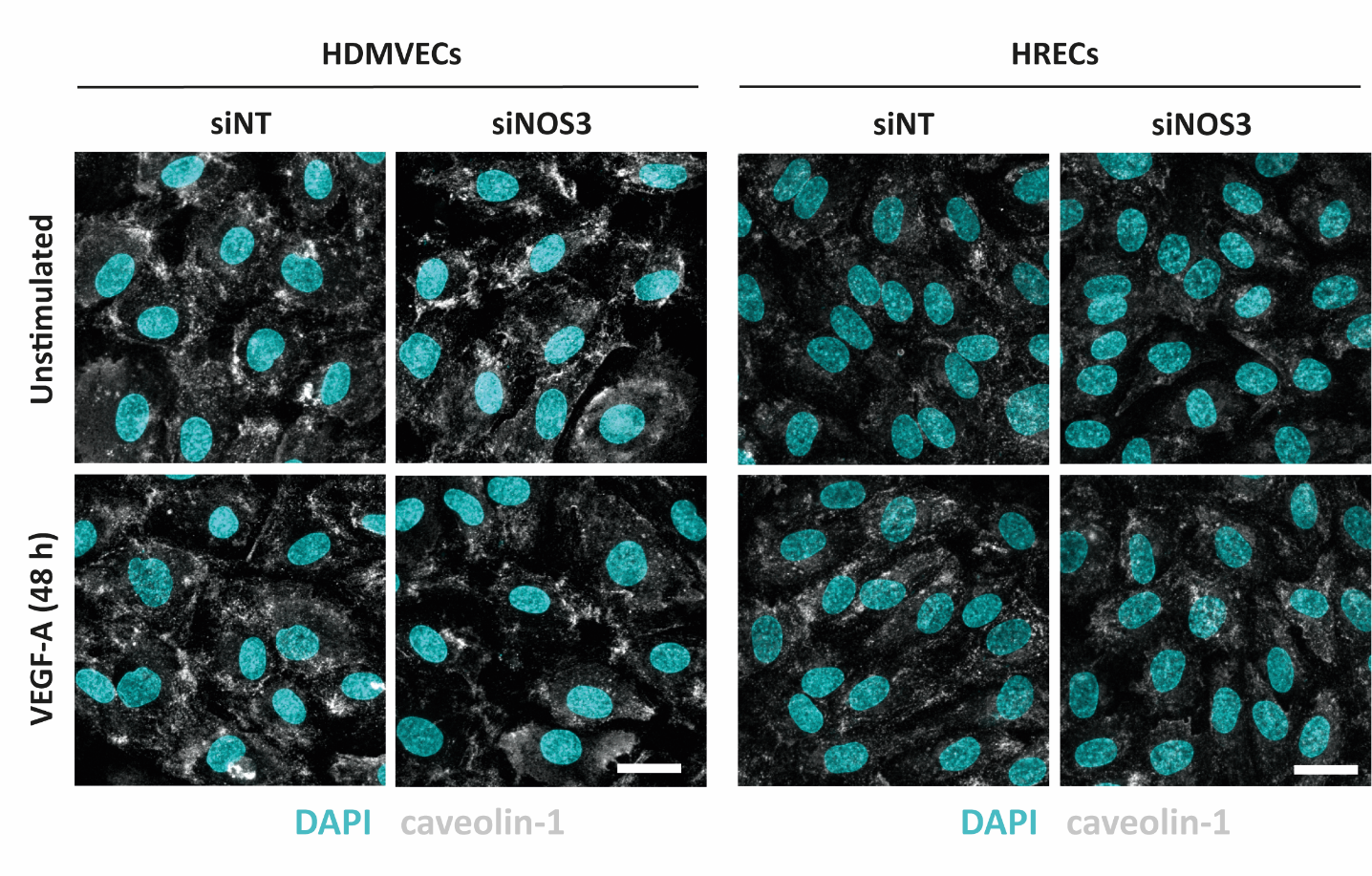
**

**Supplementary Fig. S14. CAV1 immunostaining in siNT- and si*NOS3*-treated HDMVECs and HRECs.**Representative images of CAV1 immunofluorescence staining in siNT- and si*NOS3*-treated HDMVECs and HRECs in the presence or absence of VEGF (25 ng/ml for 48 h) stimulation, in *n* = 2 independent experiments.

**Supplementary Table S1. Primer details of tested genes**. Gene nomenclature, GenBank accession code, primer sequences, and predicted size (in base pairs) and melting temperature (Tm) of the amplified products (in ˚C).

| **Human** | | | | | |
| --- | --- | --- | --- | --- | --- |
| **Gene** | **GenBank** | **Forward primer** | **Reverse Primer** | **Size(bp)** | **Tm (°C)** |
| CAV1 | NM_001753.5 | ACCTCAACGATGACGTGGTCAAGA | TGGTGAAGCTGGCCTTCCAAA | 99 | 78 |
| CHD5 | NM_001795 | CACCACGAAACGTGAAGTTCAAATTC | ACTGTGATGTTGGCCGTGTTATCG | 91 | 76 |
| CLDN5 | NM_003277 | ACCTGTTTTGCGGGCTGCTG | CCAGCTCGTACTTCTGCGACA | 108 | 84 |
| CLTC | NM_004859.4 | CATGGATTTTGCCATGCCCT | CATCAACTGGGGCTGACCATA | 148 | 78 |
| CTNNB1 | NM_001904 | TGTTCCGAATGTCTGAGGACAAGC | CAGCAGTCTCATTCCAAGCCATTG | 108 | 79 |
| DNM1 | NM_004408.4 | GAGATGGAGCGCATCGTGACCA | TGAAGTCCTCATGGTTGGTGTTCA | 118 | 83 |
| DNM2 | NM_004945.4 | TCCGTGACCTCATGCCAAAGAC | CCAGCAGCTCATGGTGGATGAA | 78 | 79 |
| EDN1 | NM_001955.5 | AACCCGCAGCGCTTTGA | GAGCTCAGCGCCTAAGACTG | 177 | 79 |
| FLOT1 | NM_005803.4 | CTCAACACACTGACCCTCAATGTCAA | TTTACCTGGGCAATGCCAGTGACT | 89 | 79 |
| FLOT2 | NM_004475.3 | CAGTGGAGCAGATTTATCAGGACCG | TTTGTCATACACGTCCTTGATGGTGA | 125 | 83 |
| FLT1 | NM_002019.4 | CCACCTCCATGTTTGATGACTACC | CAGCCCCGACTCCTTACTTTTACTG | 149 | 83 |
| FLT4 | NM_002020.5 | GCCAGGTATTACAACTGGGTGTCCT | TCTGGTTGTCCACAGAGCCTTTGT | 136 | 83 |
| HMBS | NM_000190.4 | GGCAATGCGGCTGCAA | GGGTACCCACGCGAATCAC | 64 | 79 |
| KDR | NM_002253.2 | CCAGATGACAACCAGACGGACAG | GGCACCATTCCACCAAAAGATG | 104 | 77 |
| MFSD2A | NM_001136493.2 | GCCTTATGCCCTGGATCATCTTC | TCAAAGAGGCAATAGAAAAGCAGGT | 124 | 82 |
| NOS1 | NM_000620.5 | TGTCGTCCTCAACAACCCAT | TGCATCCCGTTTCCAATGTG | 179 | 87 |
| NOS2 | AF049656.1 | CCTTCCGAAGTTTCTGGCAGCA | TCATGCGGCCTCCTTTGAGC | 69 | 81 |
| NOS3 | NM_000603.5 | TGGCTGTCTGCATGGACCTG | TCCACGATGGTGACTTTGGCT | 121 | 83 |
| NRP1 | NM_003873.7 | GCCTGCAACTTGGGAAACTGG | CCTTGGTTGGATGATGTGATCTGG | 138 | 78 |
| NRP2 | NM_003872.3 | GGAGCCCTGTGGTTGGATGTATG | TCATCTGGAAACGTCCGGTCGT | 93 | 83 |
| NSF | NM_006178.4 | AAGGCCCTCCTCACAGTGGGAA | TCATGGCCTGACATTTGGCTGT | 132 | 79 |
| OCLN | NM_002538 | CTCCCGTTTGGATAAAGAATTGG | TGCAGATCCCTTCACTTGCTTC | 103 | 76 |
| PACSIN2 | NM_007229.3 | ATGACTATGAGGGGCAGGAGCATG | TCCTCGTCCTCCATCTTGGTCAG | 76 | 80 |
| PLVAP | NM_031310.3 | GCTGCGGAAGGAACGAGACAAC | GGCATCATCGGCTGCGACTT | 138 | 85 |
| SNAP23 | NM_003825.3 | ATGAGTCTCTGGAAAGTACGAGG | CCACAGCATTTGTTGAGTTCTG | 190 | 79 |
| TJP1 | NM 003257 | ATTCTTCAAAGGGAAAGCCTCCTGA | GCATACTGCGAGGGCAATGGA | 115 | 81 |
| VAMP2 | NM_014232.3 | TGAGGGTGAACGTGGACAAGGTC | GAGGATGATGGCGCAAATCACTC | 185 | 84 |
| VAMP3 | NM_004781.4 | GGAAGAATTGCAAGATGTGGGC | TTGAGTTCCGCTGGTTCTTCA | 104 | 77 |
| VAMP8 | NM_003761.5 | AATGATCGTGTGCGGAACCT | TGAAGTGCTCAGATGTGGCT | 148 | 82 |
| VEGF-A | NM_001025366.3 | GGCAGAAGGAGGAGGGCAGAAT | CACCAGGGTCTCGATTGGATGG | 91 | 80 |
| YWHAZ | NM_003406.4 | ACTTTTGGTACATTGTGGCTTCAA | CCGCCAGGACAAACCAGTAT | 94 | 77 |
| **Bovine** | | | | | |
| **Gene** | **GenBank** | **Forward primer** | **Reverse Primer** | **Size (bp)** | **Tm (°C)** |
| HMBS | NM_001046207.1 | ATGGGCAACTGTACCTGACTGG | CCATCTTCTTGCTGAACAGGGA | 106 | 81 |
| MFSD2A | NM_001101959.1 | CCTCATGCCCTGGATCACCTT | CGTGACCAGCGTTTCAAAGAGG | 136 | 84 |
| PLVAP | NM_001035353.2 | GAAGCGTGAGACTGAGCACCTCAA | CCAGGAATCGTCAACTGCGACTTC | 96 | 81 |
| YWHAZ | NM_174814.2 | CAAGCATACCAAGAAGCATTTGA | GGGCCAGACCCAGTCTGA | 76 | 75 |
